# Supplementary material for: RANKL regulates male reproductive function
Source: Nat Commun. 2021 Apr 23;12:2450. doi: 10.1038/s41467-021-22734-8 (PMC8065035; doi:10.1038/s41467-021-22734-8)
Supplement: Supplementary file 1 — Supplementary Information [file 41467_2021_22734_MOESM1_ESM.pdf]

Supplementary Material

RANKL regulates male reproductive function

Martin Blomberg Jensen, Christine Hjorth Andreassen, Anne Jørgensen, John Erik Nielsen, Li Juel Mortensen, Ida Marie Boisen, Peter Schwarz, Jorma Toppari, Roland Baron, Beate Lanske, and Anders Juul

Supplementary Fig. 1

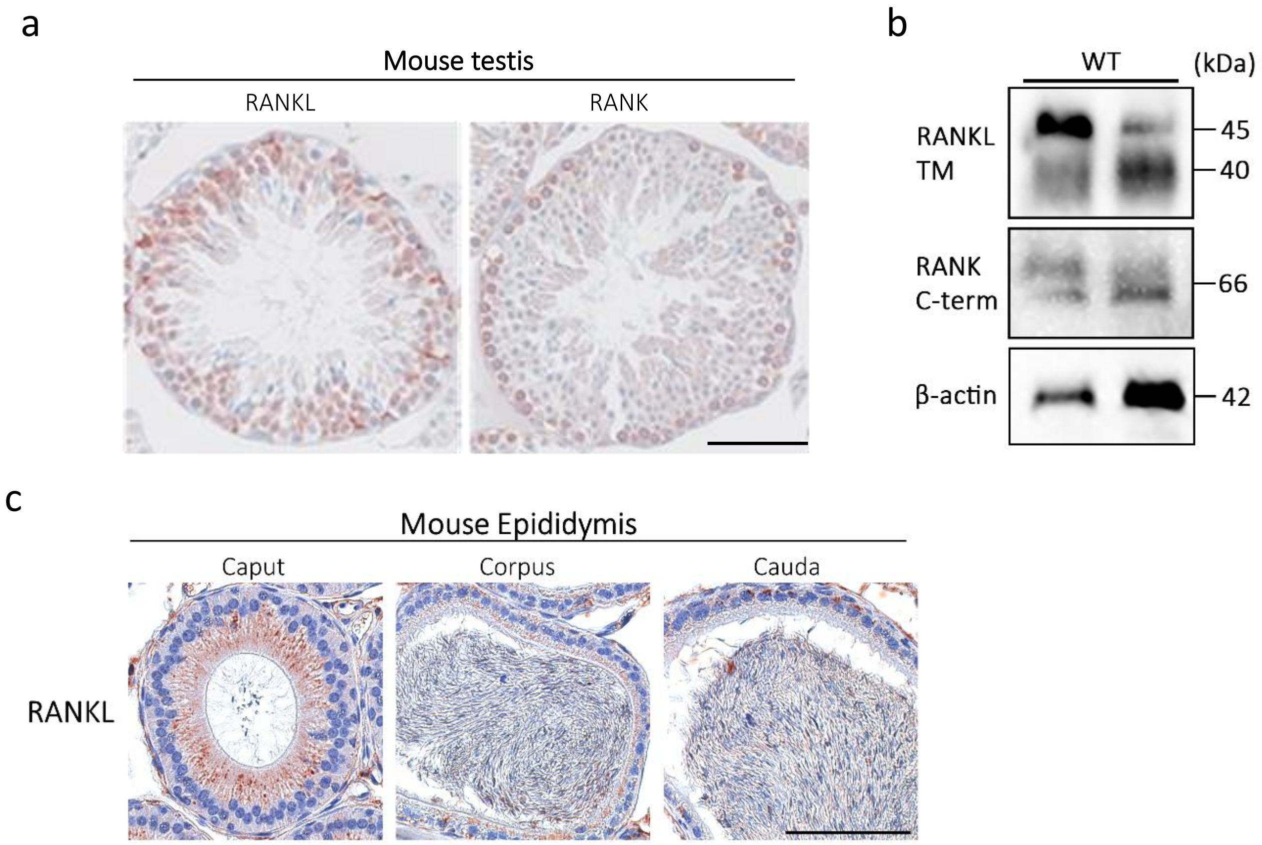

Supplementary Figure 1. **Expression of RANKL and RANK in the testis and epididymis of wildtype mice.** (**a**) IHC of RANKL (sc-9073) and RANK (sc-9072) in testis from wildtype mice (Swiss)[1,2]. (**b**) Western blot from wildtype mice (Swiss) with RANKL (sc-9073) and RANK (sc-9072). β-actin was used as a loading control. (**c**) IHC detection of RANKL (sc-9073) in the three compartments (caput, corpus, and cauda) of mouse epididymis. Counterstaining with Mayer's hematoxylin. Scale bars correspond to 50 µm. Antibody designations are specified in Supplementary Table 4. For micrographs (a-c) tissue from at least three different mice was analyzed. Abbreviations: WT, wildtype; TM, transmembrane; C-term, C-terminal. Related to Figure 1 and 2.

Supplementary Fig. 2

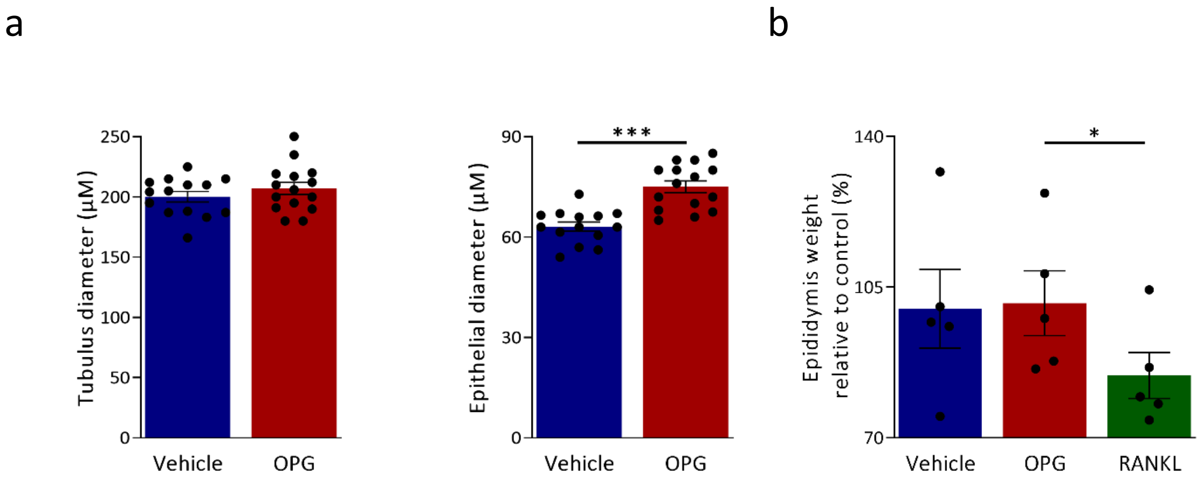

Supplementary Figure 2. Injection of OPG and RANKL into wildtype mice. (**a**) Reproductive endpoints following treatment with OPG (1 mg/kg x 2 times weekly, red) or vehicle (blue) for 2 weeks (tubule diameter and germ cell layer thickness) determined using NanoZoomer 2.0 HT and NDP.view2 Software (Hamamatsu Photonics) on cross-sections of stage VII-VIII tubules, (right panel; $p<0.0001$). (**b**) Epididymis weight after 2 weeks of RANKL (50 µg/kg x 2 times weekly, green), OPG (1 mg/kg x 2 times weekly, red), or vehicle treatment (blue), ($p=0.0464$). All mice were 8 weeks at initiation of dosing. Data presented individually and as mean ± SEM (a, b) with n (vehicle/OPG) = 14/15 (a) and n = 5 (b) in each group. Statistical test: Student's t-test (with * $p<0.05$, *** $p<0.001$. Related to Figure 1.

Supplementary Fig. 3

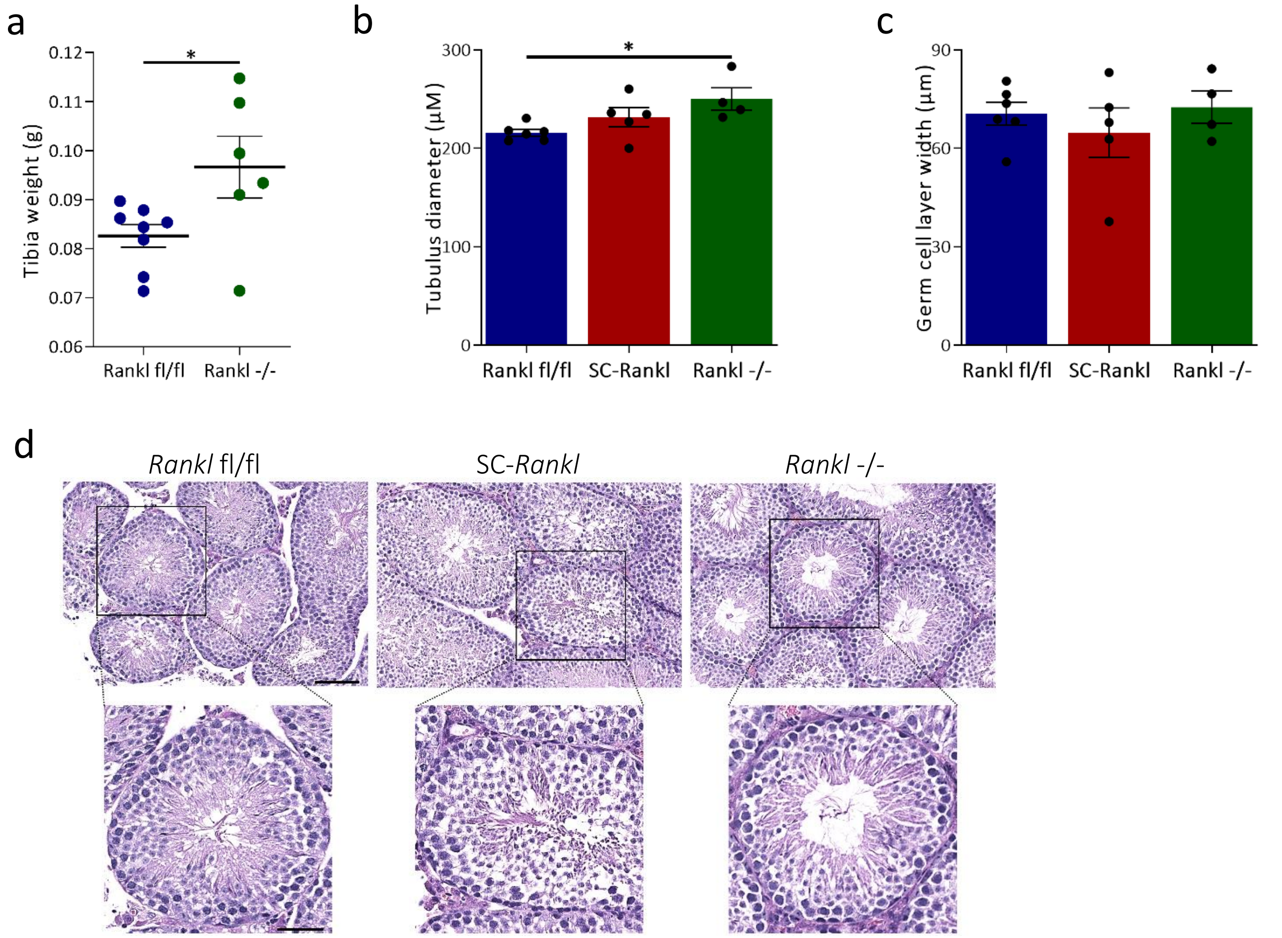

Supplementary Figure 3. Tibia weight and histological evaluation of seminiferous tubules in *Rankl* fl/fl, SC-*Rankl*, and *Rankl* -/- mice. (**a**) Tibia mass in *Rankl* fl/fl (blue) and *Rankl* -/- (green) mice, (p=0.038). Tubule diameter (**b**) and germ cell epithelial width (**c**) was determined using NanoZoomer 2.0 HT and NDP.view2 Software (Hamamatsu Photonics) on cross-sections of HE stained stage VII tubules from *Rankl* fl/fl (blue), SC-*Rankl* (red), and *Rankl* -/- mice (green), (b; p=0.29, p=0.021). (**d**) HE-staining of testis from *Rankl* fl/fl, SC-*Rankl*, and *Rankl* -/- mice. Scale bars correspond to 100 µm for low magnification (upper) and 50 µm for high magnification (lower) images. For micrographs (d) tissue from at least three different mice pr. phenotype was analyzed. Data presented individually and as mean ± SEM with n (*Rankl fl/fl*/*Rankl -/-*) = 8/6 (a) and n (*Rankl fl/fl*/*SC-Rankl*/*Rankl -/-*) = 6/5/4 (b, c). Statistical tests: Student's t-test (a) or ANOVA with Dunnett's test to adjust for multiple comparisons (b) with * p<0.05. Related to Figure 2.

Supplementary Fig. 4

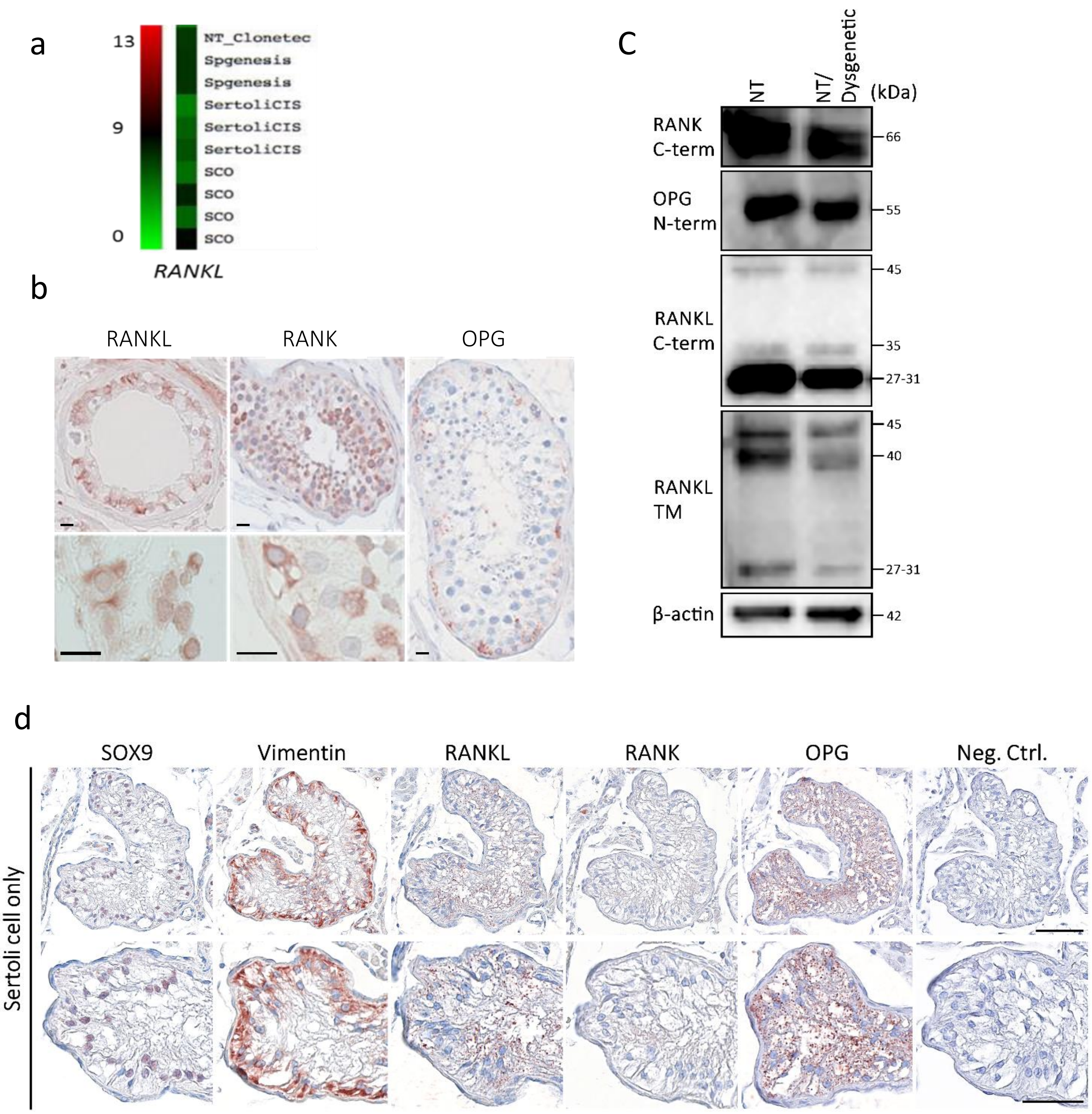

Supplementary Information 4. Expression of RANKL, RANK, and OPG in human testis specimens. (**a**) Microarray of micro-dissected human specimens showing *RANKL* expression. (**b**) IHC of RANKL (sc-9073), RANK (sc-9072), and OPG (sc-8468) in human formalin fixed testis. Top, Testis specimens with some atrophy and spermatogenic arrest. Below, higher magnification showing cytoplasmic staining of RANKL in Sertoli cells and RANK in spermatogonia and spermatocytes. (**c**) Western blot of RANK (sc-9072), OPG (sc-8468), and RANKL (top, ab9957; bottom, sc-907, bottom) from normal testis (NT) and testis with atrophy, spermatogenic arrest and tubules with carcinoma *in situ* (NT/dysgenic). Soluble RANKL is detected at 27-31 kDa and full-length RANKL is detected at 40-45 kDa both in glycosylated isoforms and unglycosylated full-length RANKL is detected at 35 kDa. β-actin was used as a loading control. Antibody designations are specified in Supplementary Table 4. (**d**) IHC of RANKL (sc-7628), RANK (sc-9072), OPG (sc-8468), and Sertoli cell markers

SOX9 (ab5535) and Vimentin (sc-373717) in tubules with Sertoli cell-only syndrome. Negative control is staining without addition of primary antibody. Scale bars correspond to 25 µm (a) and 100 µm (low magnification) or 50 µm (high magnification) (d). For micrographs (b-d) tissue from at least three different human specimens was analyzed. Counterstaining with Mayer's hematoxylin. Abbreviations: NT, normal tissue; Spgenesis, all cells inside seminiferous tubules; Sertoli CIS, Sertoli cells from tubules with carcinoma *in situ*; SCO, Sertoli cell only testis with no germ cells; C-term, C-terminal; N-term, N-terminal; TM, transmembrane; Neg. Ctrl, Negative control without primary antibody. Related to Figure 3.

59 **Supplementary Fig. 5**

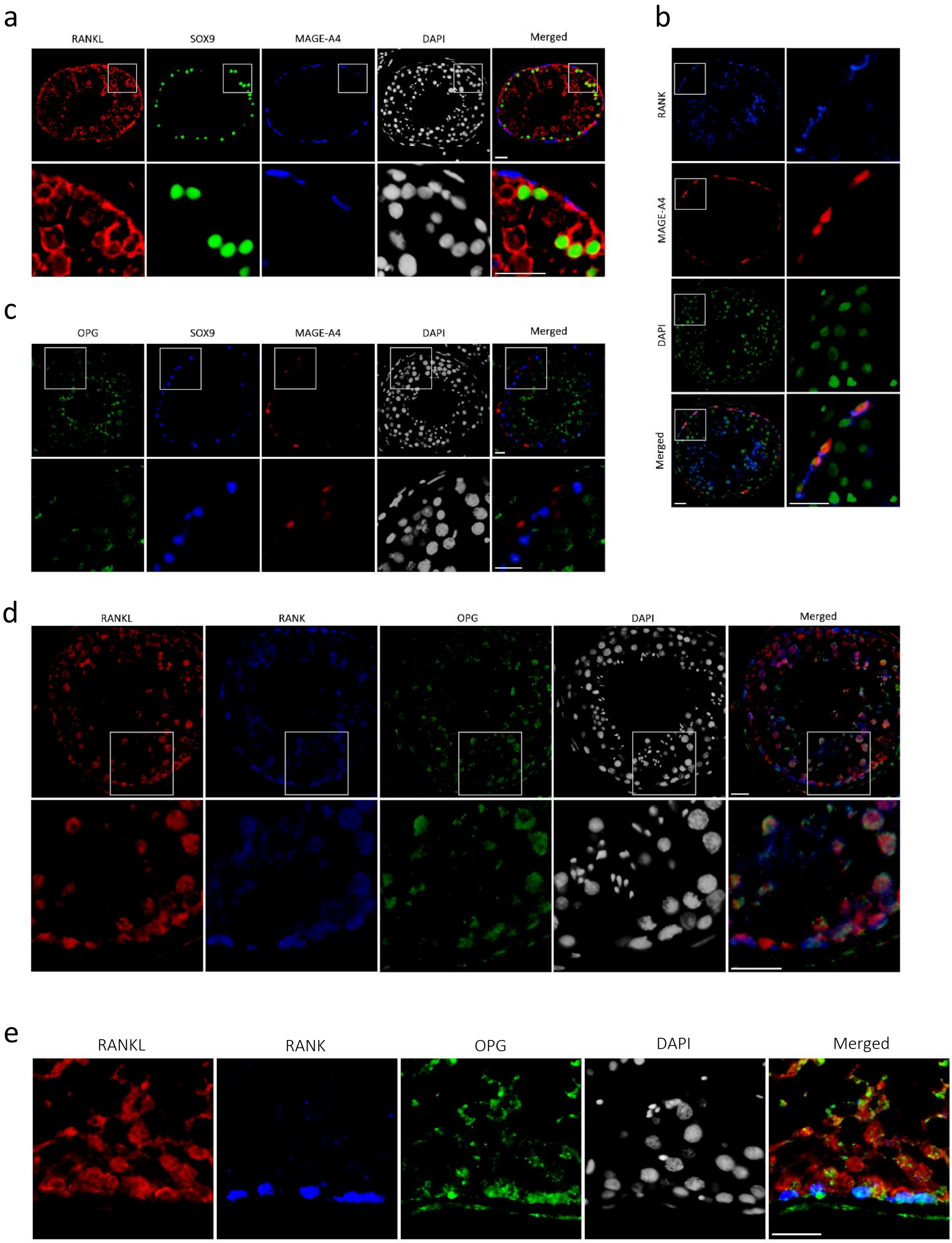

60

Supplementary Figure S5. Expression of RANKL, RANK, and OPG in formalin-fixed human testis tissue. (**a**) Triple immunofluorescence with RANKL (sc-7628, red), SOX9 (Sertoli cell marker, green), MAGE-A4 (spermatogonia/germ cell marker, blue), and DAPI (grey) in normal testis. (**b**) RANK (HPA0277728, blue), MAGE-A4 (germ cell marker, red), and DAPI (green) in normal testis. (**c**) Triple immunofluorescence with OPG (sc-21038, green), SOX9 (blue), and MAGE-A4 (red) in normal testis. (**d**) Triple immunofluorescence with RANKL (sc-7628, red), RANK (HPA0277728, blue), OPG (sc-21038, green), and DAPI (grey) in normal testis. (**e**) High magnification of triple immunofluorescence with RANKL (sc-7628, red), RANK (HPA0277728, blue), OPG (sc-21038, green), and DAPI (grey) in normal testis. Scale bars correspond to 25 µm. Antibody designations are specified in Supplementary Table 4. For micrographs (a-e) tissue from at least three different human specimens was analyzed. Related to Figure 3.

Supplementary Fig. 6

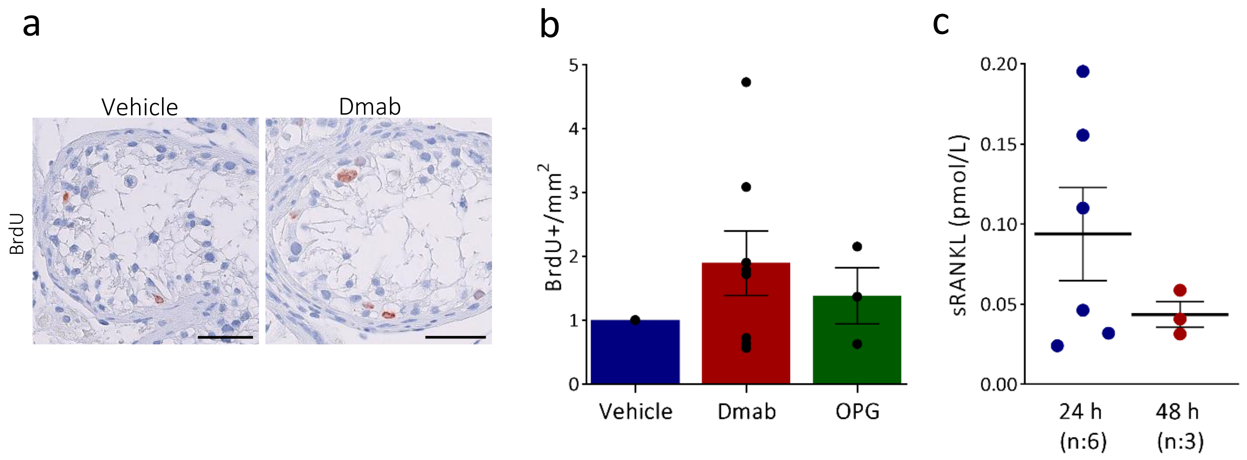

Supplementary Figure 6. The effects of RANKL inhibition on germ cell proliferation and secretion of sRANKL. (**a**) Testicular tissue with spermatogenic arrest was cultured *ex vivo* and treated with Denosumab (100 ng/ml) or vehicle for 48 hours. Proliferation was investigated by BrdU incorporation. Counterstaining with Mayer's hematoxylin. Scale bars correspond to 50 µm. (**b**) Number of proliferating cells in *ex vivo* testis culture after vehicle (blue), Denosumab (100 ng/ml, red), or OPG (50 ng/ml, green) treatment determined by BrdU positive cells per area. (**c**) Measurement of sRANKL in the media from *ex vivo* testis cultures following treatment with vehicle after 24 (blue) and 48 (red) hours. Data presented individually and as mean ± SEM, with n (vehicle/Dmab/OPG) = 8/8/3 (b) and n (24h/48h) = 6/3 (c). Antibody designations are specified in Supplementary Table 4. For micrographs (a) tissue from at least three different *ex vivo* cultures was analyzed. Abbreviations: Dmab, Denosumab; BrdU, bromodeoxyuridine; h, hours. Related to Figure 3.

Supplementary Fig. 7

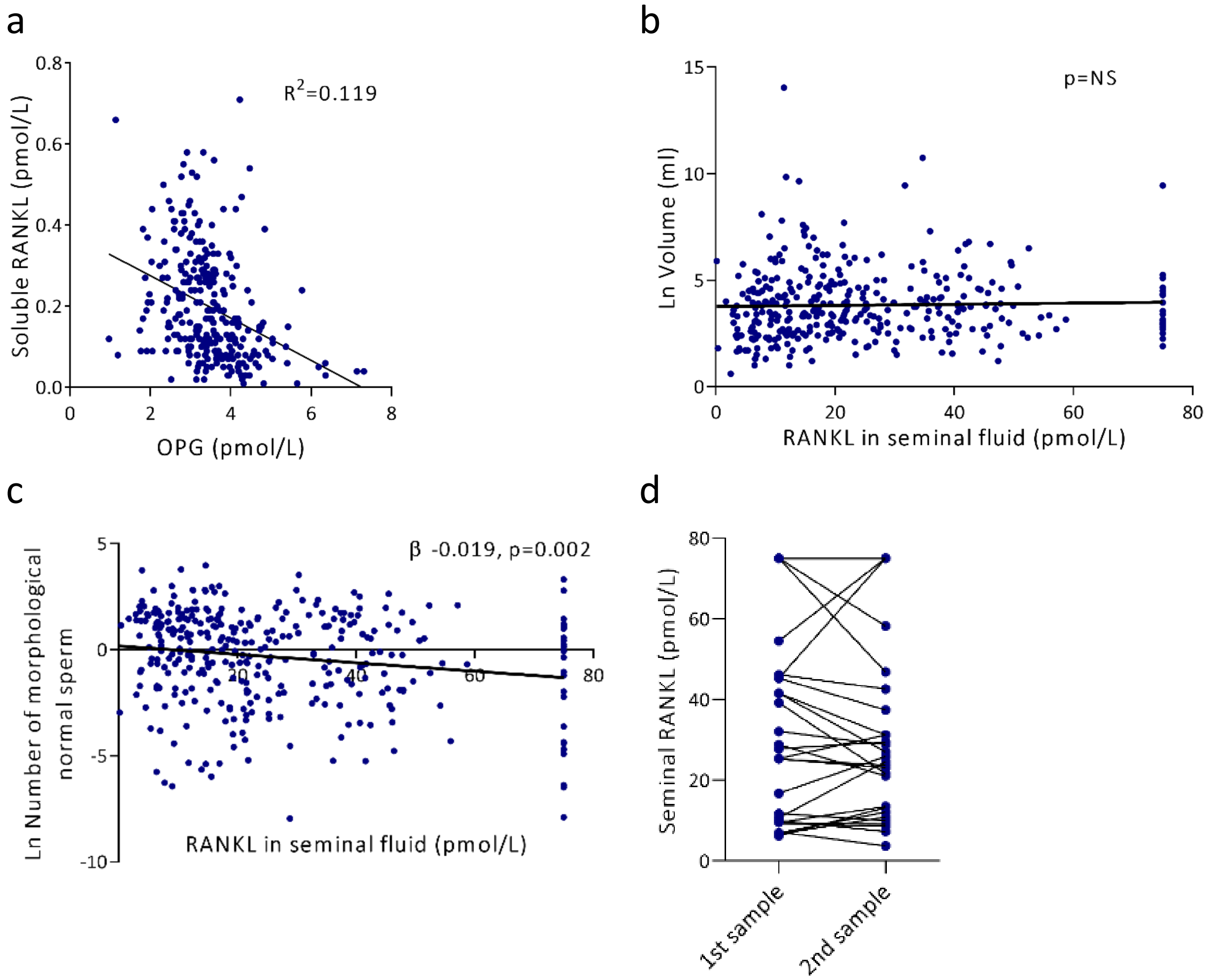

Supplementary Figure 7. **Measurements of OPG in serum and soluble RANKL in serum and seminal fluid**. (**a**) Correlation between soluble RANKL (pmol/L) and OPG (pmol/L) in serum. (**b**) Ejaculation volume and seminal RANKL levels in a pooled linear regression model of all healthy and infertile men. (**c**) Number of morphological normal sperm and seminal RANKL levels in a pooled linear regression model of all healthy and infertile men. (**d**) Repeated measurements of seminal RANKL levels in 31 infertile men with approximately 14 days between sample collection. No statistical difference between seminal RANKL levels from the first to second sample was found (p=0.84). Data presented as individual values with n = 282 (a), n = 476 (b), n = 469 (c), and n = 31 (d). Statistical tests: Linear regression model (b, c) and two-sided paired Student's t-test (d). All beta and p-values are adjusted for time of abstinence. Abbreviations: Ln, natural logarithm; NS, not significant. Related to figure 5.

Supplementary Fig. 8

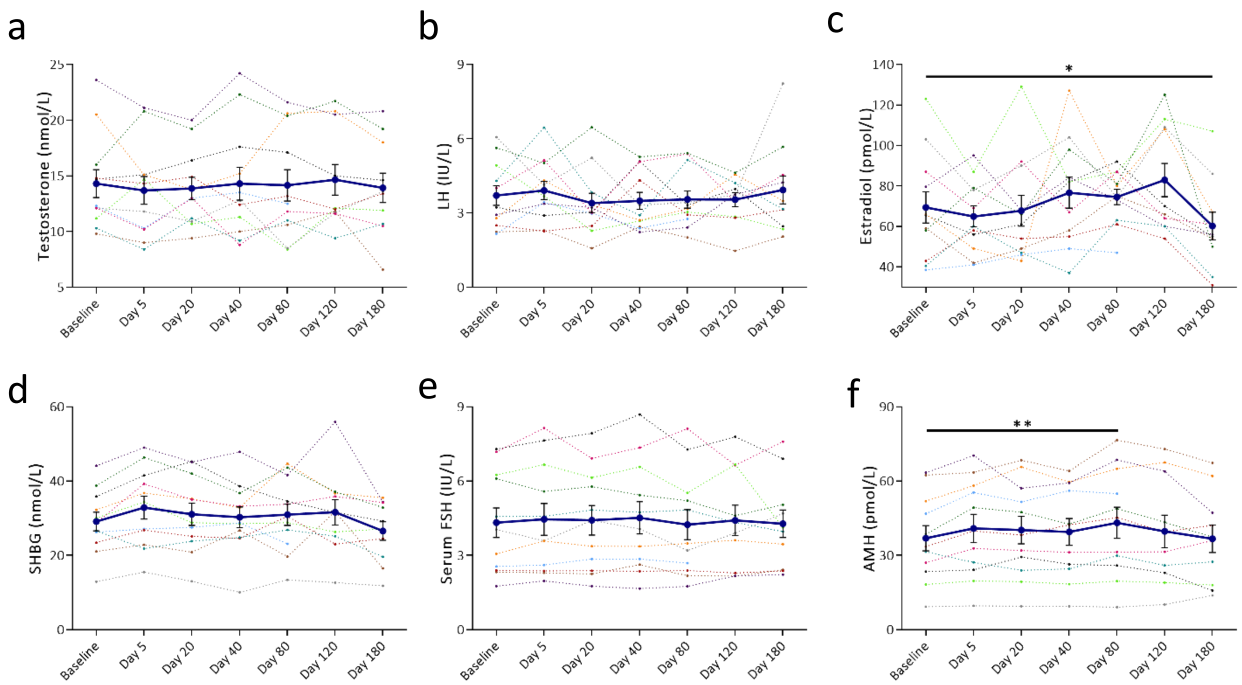

Supplementary Figure 8. Changes in sex steroids and reproductive hormones. (**a**) Testosterone (nmol/L), (**b**) LH (IU/L), (**c**) Estradiol (pmol/L), (**d**) SHBG (nmol/L), (**e**) FSH (IU/L), and (**f**) AMH (pmol/L), measured 5-180 days after treatment with Denosumab (60 mg) once in 10 infertile men, (c; p=0.04, f; p=0.004). All hormones and steroids are presented as individual raw values (dotted lines) combined with mean ± SEM (blue). Note, y-axes and x-axes do not intersect at 0 (a, c). Statistical test: Paired two-sided Student's t-test (c, f) with $*p<0.05$, $**p<0.01$. Abbreviations: SHBG, Sex hormone-binding globulin; LH, Luteinizing hormone; FSH, Follicle-stimulating hormone; AMH, Anti-Müllerian hormone. Related to Figure 6.

Supplementary Fig. 9

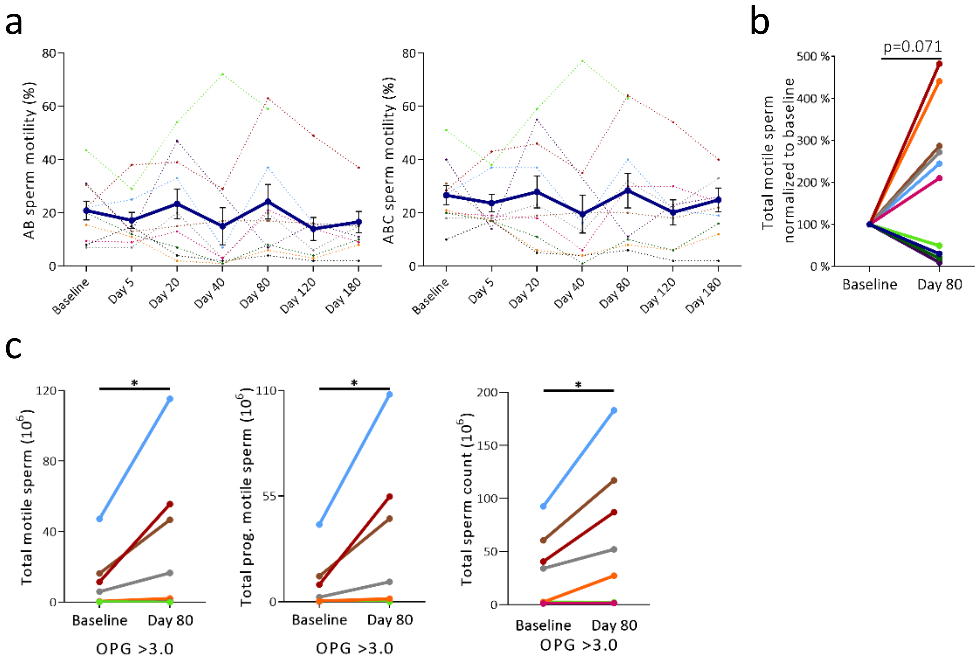

Supplementary Figure 9. Changes in semen quality over time after Denosumab (60 mg) injection in infertile men. (**a**) AB (progressive) sperm motility (%) and ABC (total) sperm motility (%) presented from baseline to day 180. (**b**) Individual change in total motile sperm normalized to baseline shown at baseline and day 80. (**c**) Total motile sperm (left), total progressive motile sperm (middle), and total sperm count (right) presented as raw values at baseline and day 80 for men with OPG >3.0 pmol/L, (left panel: p=0.04, middle panel: p=0.02, right panel: p=0.02). Baseline is calculated as average of two semen samples delivered prior to treatment start. Total motile sperm and total progressive motile sperm were log2 transformed for statistical analysis. Data presented as raw data for individual patients (dotted lines) and as mean ± SEM (blue) (a) or as individual values (b, c). Statistical test: Paired two-sided Student’s t-test (logarithmic transformation) (b, c) with * p<0.05. Abbreviations: OPG, Osteoprotegerin. Related to Figure 6.

Supplementary Fig. 10

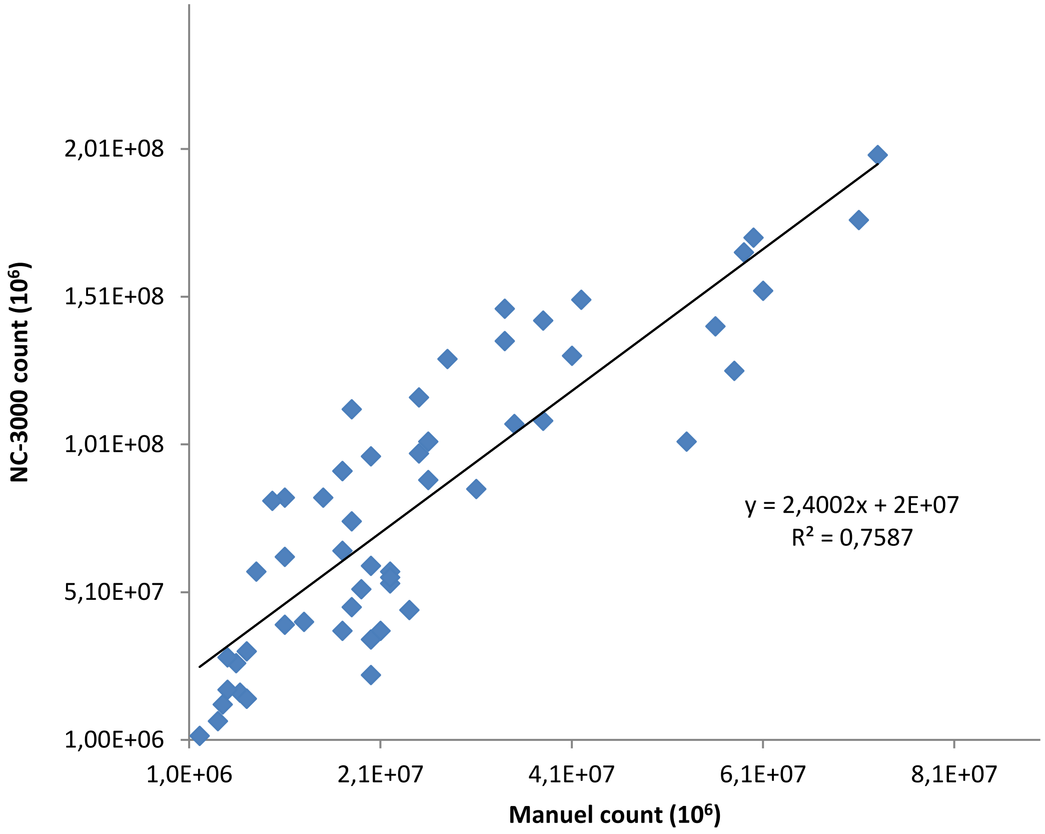

Supplementary Figure 10. Validation of sperm count in mice. Sperm count was determined manually with the technician blinded to treatment and genotype and was conducted exclusively by one person. The technician only counted sperm heads as the tail was separated from the head in many sperm. 55 sperm samples assessed manually were also counted automatically using NC-3000 (Chemometec). NC-3000 overestimates the number of cells, but a strong correlation between manual and automatic assessment was found (R^2: 0.76). Data presented as individual values. Related to Figure 2 and Materials and Methods.

Supplementary Methods 1

Supplementary Methods 1. Generation of mice with Sertoli specific and global RANKL-deficiency

Sertoli-specific (AMHCre;Rankl$^{fl/fl}$) and global (VasaCre;Rankl$^{fl/fl}$),) knock-out mice.

To determine the tissue specific function of RANKL-deficiency *in vivo* we obtained *Rankl$^{fl/fl}$* mice *(Cat# B6.129-Tnfsf11* from the Jackson Laboratory. We first crossed *Rankl$^{fl/fl}$* with *AMHCre-Tg* mice (kind gift from Prof. Jorma Toppari, backcrossed onto C57BL/6) to generate a Sertoli-specific RANKL-deficient mouse line [3]. Cre expression was validated by crossing the *Rankl$^{fl/fl}$* mice with mice carrying a *Rosa26-tdtomato*-allele. Our subsequent aims were to generate mice with germ cell specific and global RANKL-deficiency. However, when we crossed *Rankl$^{fl/fl}$* with *VasaCre-Tg* mice (*Cat# B6.FVB-Tg(Ddx4-cre)1Dcas/KnwJ*) obtained from the Jackson Laboratory. The generated *VasaCre;Rankl$^{fl/fl}$* pups did not have the germ cell specific knock out of *Rankl* that was expected. We show that *VasaCre;Rankl$^{fl/fl}$* pups had global deletion of *RANKL* and not exclusively in the germ cells. It is known that offspring of female *Vasa-Cre* mice will have active Cre globally and this occurs also in 10-15% of pups inheriting the *Cre* from the father. Genotyping was complicated by interference between presence of the *Vasa-Cre* allele and genotyping of the floxed *Rankl* allele. When mice harboring the *Vasa-Cre* allele were genotyped (primer 1+2) then the *Rankl* alleles exclusively showed presence of the wildtype alleles. By applying a new primer set that was able to show deletion of *Rankl* systemically we could generate the expected genotype, which enabled us to control the breeding (see explanation below).

We used the primers listed in the table below. The binding site for primer 2 is lost when the floxed allele is deleted, thus primer 1+2 will produce no bands in a homozygous null mouse. Primer 4 was used to detect the original targeted allele prior to deletion of the neo selection cassette. The most likely explanation is that the *Vasa-Cre* is active in the male germ cells and that sufficient Cre activity remains in the fertilized egg to cause deletion of RANKL in the developing offspring. Therefore, we may be unable to detect the floxed allele because it has been recombined, although this cannot explain why we see a wildtype band. An additional primer set was used and *Rankl* floxed primers 1+3 produce a 280 bp band for a deleted allele. If this shows up in the tail DNA of the *Vasa-Cre*-positive mice, then germline deletion has taken place. When we use primer 1+2 or primer 1+3 on pups from the *Vasa-Cre* male obtained from Jackson that are *Rankl* wt/wt mated with female *Rankl$^{fl/fl}$* then the results were as follows: Using the *Rankl* floxed 1+2 primers an expected band at 108 bp for the wildtype allele showed and a band at 251 bp for the floxed allele, except for *Vasa-Cre* positive pups that only show the 108 bp fragment. Primer set 1+3 showed as expected no band in all pups except for a 280 bp band in *VasaCre* positive pups, which demonstrates global deletion. To validate this global RANKL deletion we breed the mice with wildtype and *Rankl$^{fl/fl}$* mice that proved presence of deleted alleles in these mice because RANKL floxed 1+2 showed heterozygotes when breeding with wildtype mice and exclusively the 251 bp band when breeding with *Rankl$^{fl/fl}$*. Noteworthy, in 2 % of breedings with paternal inherited *Vasa-Cre* offspring did not have global deletion and became germ cell specific. Primer set 1+3 was able to detect this because it showed no deletion and thus no band at 280 bp. Vasa-Cre activity was validated by crossing the mice with *Rosa26-tdtomato*-mice and further supported by qPCR and WB from different organs.

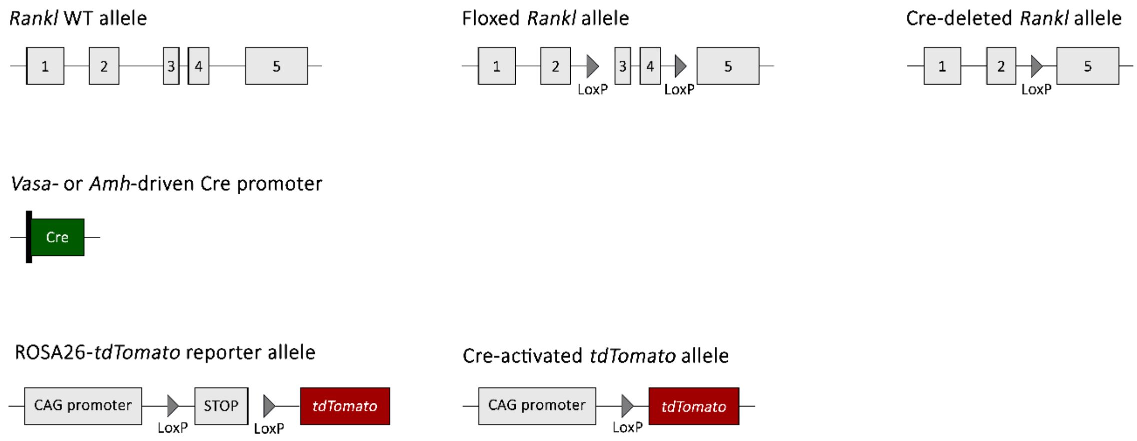

Primers used for genotyping RANKL Sertoli deficient and global deficient mice

Gene	Primer name	Primer sequence 5’-3’
Rankl (Tnfsf11)	Rankl-flox 1	CTGGGAGCGCAGGTTAAATA
Rankl (Tnfsf11)	Rankl-flox 2	GCCAATAATTAAAATACTGCAGGAAA
Rankl (Tnfsf11)	Rankl-flox 3	CTCAGCTTCCAGAGGACTGC
Rankl (Tnfsf11)	Rankl-flox 4	GTGGGCTCTATGGCTTCTGA
Vasa	VasaCre-1	CACGTGCAGCCGTTTAAGCCGCGT
Vasa	VasaCre -4	TTCCCATTCTAAACAACACCCTGAA
Amh	AMHcre-1	CCTGGAAAATGCTTCTGTCCG
Amh	AMHcre-4	CAGGGTGTTATAAGCAATCCC

**RANKL primer pairs**		**Amplicon size**	
	**1 + 2**	**1 + 3**	**4 + 2**
**Target**			
Wild type locus	108 bp	1278 bp	no product
Targeted locus	1965 bp	3261 bp	213 bp
Delta neo	251 bp	1547 bp	no product
Delta exon 3-4	no product	280 bp	no product

Genotyping on tailtip-PCR

Lane 1-6 and 15-28: pups from *Vasa-Cre* + *Rankl*$^{fl/fl}$

Lane 7-14: pups from *Amh-Cre* + *Rankl*$^{fl/fl}$

Lane 29-30: Control *Rankl*$^{fl/fl}$ homozygote mother from JAX

Lane 31-32: Control *Amh-Cre* father

Lane 33-34: Control *Vasa-Cre* father
Lane 35: Negative control (H_2O)

Primer set Rankl-flox 1+2. Fragment 251 bp, floxed; 108 bp, Wt:

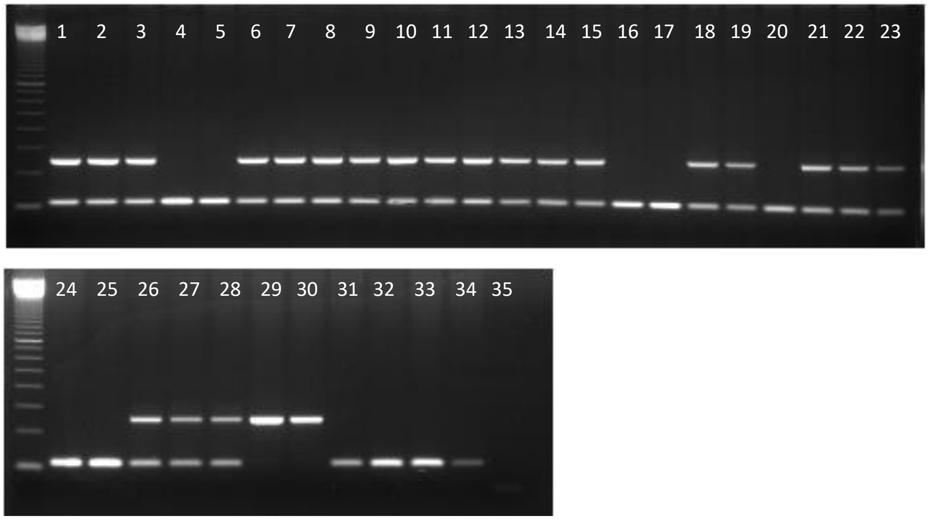

Primer set VasaCre-1 + 4. Fragment 230 bp, positive for *VasaCre*:

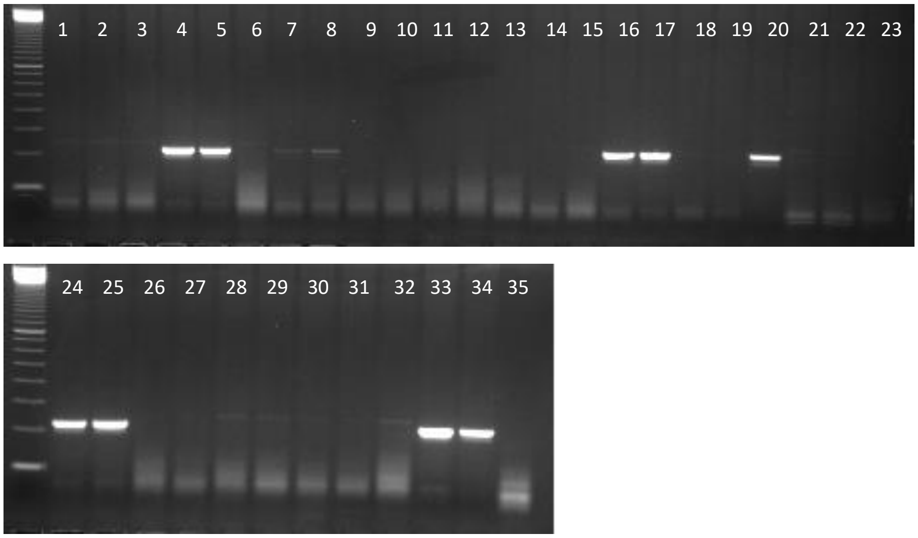

Example of pups positive for Vasa-Cre with PCR of *Rankl* floxed alleles always showing the wildtype allele only. This is wrong because we genotyped all the mothers provided by Jax and they are all homozygous for the floxed RANKL allele. Even when breeding these heterozygous male pups with *Vasa-Cre* with *Rankl*$^{fl/fl}$ we only see wildtype allele in the PCR although all these pups are either heterozygous or homozygous for the *Rankl*$^{fl/fl}$.

Example of a *Vasa-Cre* positive mice having germ cell specific *Rankl* loss rather than global loss. Mouse number 81 is *Vasa-Cre* positive, *Rankl* 1+2 shows the typical wildtype presence but *Rankl* 1+3 shows no global deletion.

This mouse has only loss of *Rankl* in the germ cells but induces global loss in his *Vasa-Cre* positive offspring while the *Vasa-Cre* negative offspring have no *Cre* activation as seen after breeding with a *Rosa26-tdtomato*-mouse.

Primer set *VasaCre*-1 + 4. Fragment 230 bp, positive for Cre:

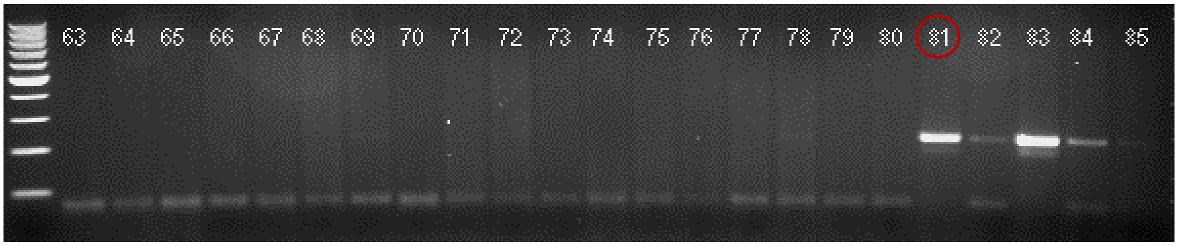

Primer set *Rankl-flox*1 + 2. Fragment 251 bp,floxed; 108 bp, Wt:

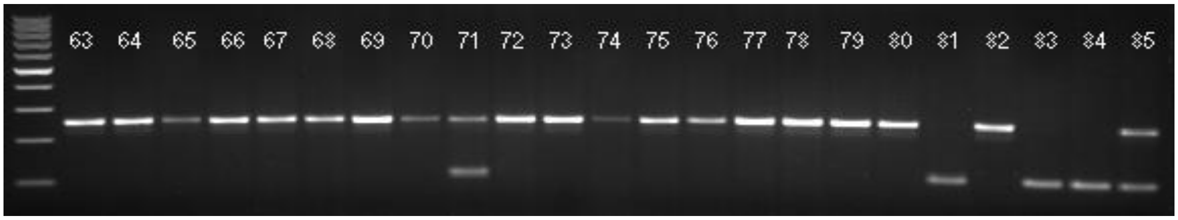

Primer set *Rankl-flox*1 + 3. Fragment 280= deleted

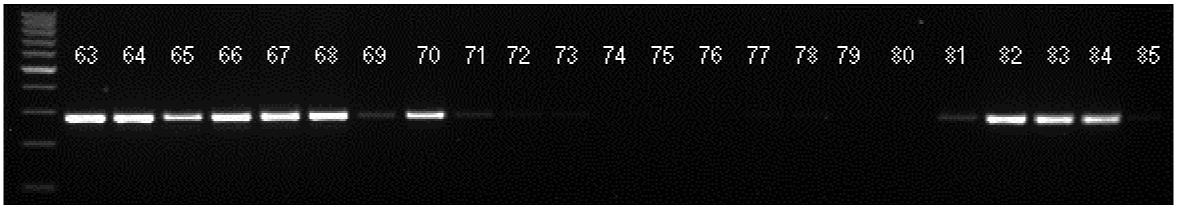

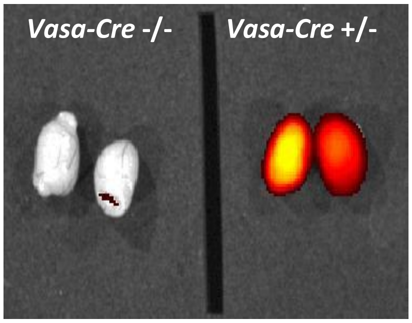

Primer set *VasaCre*-1 + 4. Fragment 230 bp, positive for *Cre*:

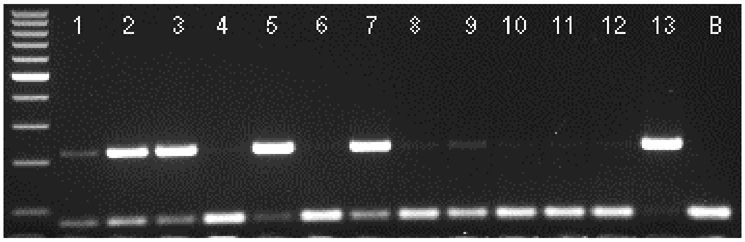

Primer set *Rankl flox*-1+2. Fragment 251 bp, floxed; 108 bp, Wt:

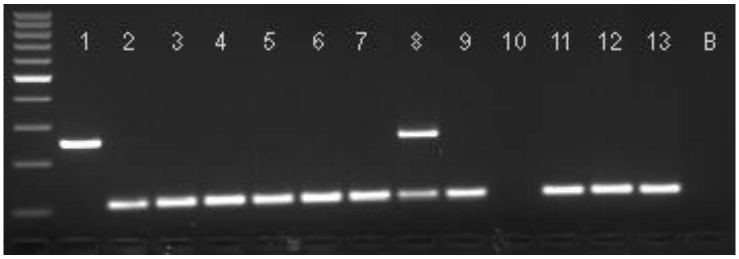

Primer set *Rankl flox*-1+3.

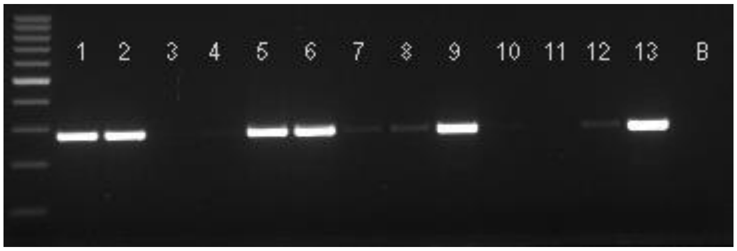

Here is an example (pup 1) where the *Vasa-Cre* positive father was bred with *Rankl*$^{fl/fl}$ females. The mouse does not express *Vasa-Cre* and it is evident that the mice got a floxed allele from the mother and a deleted allele from the father.

Supplementary Methods 2

Supplementary Methods 2. Vitamin D receptor knock-out mice. Vitamin D receptor knock-out mice were generated as described previously[1] and used to evaluate the reproductive phenotype[2]. Briefly, animals were bred in KU Leuven and received a standard rodent diet containing 1.0 % calcium and 0.7 % phosphorus. Representative groups of mice (4 *Vdr+/+*, 3 *Vdr+/-* and 6 *Vdr-/-*) were sacrificed at 10 weeks of age. Frozen and formalin fixed testicular and epididymal tissue were shipped to Copenhagen for histological and molecular analyses.

Supplementary Methods 3

Supplementary Methods 3. Detailed description of Material and Methods.

Gene expression

Gene expression levels were analyzed by quantification of cDNA with RT-qPCR. The analysis was conducted with specific primers (Table S2 and S3) that were designed to span intron-exon boundaries and all primers were tested by RT-PCR and subsequent direct sequencing (Eurofins Genomics, Germany). Briefly, cDNA was mixed with forward and reverse primers (10 pmol/µl), sterile water and QPCR Master Mix (Agilent, #600828). The PCR reaction was carried out on Mx300P Platfom (Stratagene, USA) or QuantStudio 3 (Thermo Fischer) with the following cycling conditions; 15 minutes at 95 °C, followed by 40 cycles of 15 seconds at 95 °C, and 1 minute at 62 °C. The melting curves were measured at 95 °C for 15 seconds, 62 °C for 1 minute, and finally 95 °C for 1 second, to test for correct primer binding and dissociation. All samples were run in triplicates. Expression of the gene of interest was normalized to expression of β-*2microglobulin* (*B2M*) or *Ribosomal Protein S20* (*RPS20*) by the delta-delta Ct method.

Protein purification and western blot

Protein were purified from human or mouse tissue samples or organs for western blotting. Lysis buffer was added to each sample depending on size of tissue. The tissue samples were homogenized with pellet pestles (Sigma-Aldrich, #2359947-100EA, Motor rotor from Kontes), lysed on ice and vortexed. Protein concentration was determined with the Micro BCA protein assay kit (Thermo Scientific, #23235). Protein samples were prepared for blotting by mixing 10-20 µg sample and 10% β-mercaptoethanol 2xSDS sample buffer (Sigma-Aldrich, #M6250) with equal amounts of double distilled H_2O. Samples were mixed and heated at 95 °C for 5 minutes followed by cool down. Samples were loaded on sodium dodecyl sulfate polyacrylamide gel electrophoresis gels (BioRad, #456-8096) along with 3 µl Pageruler prestained protein ladder (Thermo Scientific, #26616) and 2 µl Precesion Plus protein ladder (BioRad, #161-0376). The proteins were wet-transferred to a PVDF membrane (Biorad, #1630174) and blocked with 5% skim milk in TBS. Subsequently, primary antibody was added overnight in 1 % skim milk in 0.1 % TBS-Tween. Membranes were incubated with secondary antibody (Dako, anti-mouse, P0260, anti-rabbit P0217 or anti-goat P0160) for 1 hour. Between all incubation steps, the slides were washed with 0.1 % TBS-Tween. Visualization was performed with ECL reagent (Thermo Fischer, #34095). The results were analyzed with Chemidoc Image Lab software, version 5.2.1. Primary antibodies and dilutions are listed in Table S4 and S5.

Immunohistochemistry

Sections were deparaffinized in 2 times xylene 5 minutes each, 100%, 100%, 95%, 70% ethanol and water 20 seconds each. The staining of immunohistochemical sections were conducted on formalin fixed tissue blocks sectioned and placed on Superfrost glass slides (Thermo Fischer Scientific, #J1800AMNT), the paraffin-embedding sealed onto the glass slides in a 65° C oven for one hour and the sections were subsequently kept at 4 °C until IHC was performed. In brief, antigen retrieval was achieved by microwaving the sections in retrieval buffer. Blockade against endogenous peroxidase was performed with 0.5% (v/v) H_2O_2 in water. Sections were incubated with 2 % non-immune goat serum (Zymed Histostain kit, USA) or 0.5 % milk powder diluted in TBS. Subsequently, slides were incubated with primary antibody over night at 4 °C and 1 hour at room temperature.

The secondary antibody was added (Zymed, #956543 for mouse and rabbit antibodies, Binding Site, #ab260CUS01 for goat antibodies) and the slides were incubated for 30 minutes followed by tertiary layer (Peroxidase-conjugated Streptavidin complex, Zymed histostain kit, #956143). Between all incubation steps, the slides were washed with TBS except between blockade for unspecific binding and the primary antibody. Visualization was performed with amino ethyl carbasole (AEC) (Invitrogen, USA) for up to 20 minutes. After development the slides were washed in running water for 10 minutes and counterstained with Mayers hematoxylin. The slides were mounted with Aquamount hydrophilic substance (Merck, #108562) and 24x50 mm mounting glasses (Hounisen, #0422.2450). The tissue sections were scanned on a NanoZoomer 2.0 HT (Hamamatsu Photonics) and subsequently analyzed using the NDP.view2 Software (Hamamatsu Photonics). Primary antibodies, dilutions and retrieval buffers are listed in Table S4 and S5. For tissues fixed in GR fixative (200 mL 37% Formaldehyde, 40 mL acetic acid added to 1 L of 0.05 M phosphate buffer, pH 7.4) antigen retrival was performed with a pressure cooker. In brief, sections were placed in a pressure cooker in antigen retrieval buffer and subsequently endogenous peroxidases were blocked in 1 % H_2O_2 in methanol for 30 minutes. Sections were incubated in either 0.5 % skim milk in TBS, horse serum (ImmPRESS Kit, Vector Laboratories) or 0.5 % BSA in horse serum for 30 minutes. Sections were incubated with primary antibody diluted in blocking solution overnight at 4 °C and at room temperature for one hour. Incubation with secondary antibody (ImmPRESS Kit, Vector Laboratories, mouse #MP-7402, rabbit #MP-7401, goat #MP-7405) was carried out for 30 minutes. Between all incubation steps, the slides were washed with TBS except between blockade for unspecific binding and the primary antibody. Visualization was performed with ImmPACT DAB peroxidase (HRP) substrate (Vector Laboratories, #SK4105). The subsequent contrast staining, mounting and analysis were performed as previously described. Negative control stainings were included for both protocols (primary antibody replaced by dilution buffer) and none of the negative control slides showed staining.

Immunofluorescence

Sections were deparaffinized in 2 times xylene 5 minutes each, 100%, 100%, 95%, 70% ethanol and water 20 seconds each. Antigen retrieval was performed in a pressure cooker at 110 °C for 30 minutes in citrate buffer followed by 2 minutes in water and peroxidase blocking with 3 % H2O2 in methanol for 30 minutes. Sections were washed in TBS 2 times for 5 minutes before blocking for unspecific staining with 5 % (w/v) BSA in 20 % (v/v) horse serum (ImmPRESS Kit, Vector Laboratories) and 80 % v/v TBS for 30 minutes. This blockade was drained of with a towel before incubation over night at 4°C with the desired primary antibody diluted in the BSA horse serum TBS solution (see Table S5 for primary antibody details). Next day, the slides were washed 2 times for 5 minutes in TBS before incubation with the appropriate secondary antibody (chicken anti-mouse peroxidase (sc-2962), chicken anti-rabbit peroxidase (sc-2963) or chicken anti-goat peroxidase (sc-2961) from Santa Cruz) diluted 1:200 in the BSA horse serum TBS solution for 30 minutes. Excess antibody was removed with 2 times washing in TBS for 5 minutes before application of the desired fluorescence colour (green fluorescein NEL741E001KT, blue cyanine5 NEL745001KT or red cyanine3 NEL744E001KT from AKOYA biosciences, Marlborough, MA, USA 01752) diluted 1:50 in fluorescence kit buffer for 10 minutes, from this step the sections should be kept as dark as possible. In order to apply a second (or third) primary antibody to the sections the protocol was repeated from the pressure cooker step to the fluorescence application followed by washing with TBS for 5 minutes, counterstaining with DAPI (D3571, Molecular probes, Life Technologies Corporation, 29851

Willow Creek Road, Eugene, Oregon 97402) diluted 1:600 in TBS for 10 min, washing in TBS 2 times for 5 minutes and mounting with ProLongTM Gold antifade (P36930, Life technologies Corporation).

Supplementary Methods 4

Supplementary Information 4. *Ex vivo* model of human testis specimens.

Collection of tissue from testicular cancer patients

Testicular cancer patients orchiectomized as part of their normal treatment were recruited from the Andrology Clinic of the Department of Growth and Reproduction at Copenhagen University Hospital (Denmark) in accordance with the Helsinki Declaration and following approval from the regional ethics committee (permit number H-1-2012-007). All participants gave informed written and oral consent before orchidectomy for treatment of testicular cancer. Following orchidectomy the specimens were transported immediately after surgical removal to the Pathology Department where it was divided into tumor and macroscopically normal areas. The majority of both testicular and tumor tissue was assigned for diagnostic analysis, with the remainder allocated for research. The sample assigned for research were immediately placed in culture media (see below) and transported to the laboratory.

Ex vivo culture of human testis tissue

Ex vivo culture of human testis tissue was conducted as previously described (Jørgensen et al. 2014) with a few adjustments. In brief, within 2 hours of surgical removal of the testis the tissue was cut into 1 mm^3 fragments and was placed in 'hanging drop' cultures in 40 µl culture media. Media composition was: DMEM F12, 1 × penicillin/streptomycin, 1 × L-glutamine, 1 × insulin, transferrin and selenium (ITS) and 10% fetal bovine serum (FBS). All cell media and supplements were from GIBCO (Nærum, Denmark), except ITS (Sigma-Aldrich, Broendby, Denmark). Drops of culture media were placed on the lid of a Petri-dish (NUNC cell culture Petri-dish) and individual tissue fragments were placed into each drop, and the dish was subsequently inverted keeping the drops intact with the tissue suspended. DPBS (10 ml) was added to the bottom of the dish to prevent dehydration. Tissues were cultured at 34°C in 5% CO_2 for 24-72 hours, with complete media change every 48 hours and media from each treatment group was pooled throughout the experimental period. Tissue fragments were cultured in media containing 50 ng/ml OPG-FC (Prospecbio, cyt-266), 100 ng/ml Denosumab (Prolia, Amgen), 1 µg/ml recombinant human RANKL (R&D Systems, #6449-TEC) or vehicle (0.1 % BSA in sterile water or 0.1 % BSA in PBS). The experimental set-up included at least three replicates of tissue pieces cut from the same original tissue piece for each of the treatment groups. At the end of the culture period the tissue was fixed in formalin (30 min at room temperature, 4°C overnight) followed by paraffin embedding for histological analysis and immunohistochemistry.

BrdU incorporation

BrdU incorporation was used to determine the presence of proliferating germ cells just before the end of the *ex vivo* culture period as previously described (Jørgensen et al. 2014). In brief, BrdU labelling reagent (Invitrogen, Camarillo, CA, USA) was diluted 1:100 in culture media and tissue fragments were set-up as hanging drop cultures in BrdU-containing media for 6 h. Tissue pieces were then washed twice in PBS for 5 min followed by fixation and paraffin embedding as described above. BrdU was visualized by immunohistochemistry using a BrdU antibody in standard IHC protocol, with positive nuclear stained cells considered as proliferating.

Supplementary Table 1

	Expression pattern of human testis specimens						
Antibody designation	Spg	Spc	rSpt	eSpt	Ser	Ley	PMC
RANKL TM (n=6)	Neg	C++ to +/-	C+ to +/-	N+++	C+	C++ to +/-	C+/- to neg
RANKL C-term (n=5)	+/-	C+ to +/-	C+ to +/	Neg	Neg	C+ to +/- and neg	Neg
RANKL N-term (n=5)	Neg to +/-	C+/- to +	Neg to +	Neg to +/-	C+++	C++ to neg	C+/-
RANK C-term (n=5)	C++ to +/-	C+ to +/-	+ to +/- *	Neg to +	C+/- to neg	C+ to neg	C+/-
RANK internal (n=4)	C+++ to +/-	C+/- to neg	Neg	C++ to +	C+ to +/-	C+++ to +/-	Neg
OPG N-term 2 (n=4)	C+/-	Neg	C+/- to +	Neg	C+ to +/-	C+/- to neg	Neg to +
OPG N-term (n=4)	C+ to +/-	Neg	Neg	Neg	C++ to +/-	C+/- to neg	Neg to +

Supplementary Table 1. Table showing expression of RANKL, RANK, and OPG with 7 antibodies in human testis specimens. 3 antibodies for RANKL, 2 for OPG and 2 for RANK were tested with IHC on GR-fixed samples. (n) corresponds to the number of samples investigated with the specific antibody. The range of expression of all investigated samples is stated. The IHC score was determined with NDP.view2 software and the image settings were identical for all samples analyzed. *IHC score:* +++: strong staining in all cells of a given type in the sample. ++: staining in nearly all cells of a given type in the sample. +: weak staining overall. +/-: weak staining in limited areas. Neg: no staining. N: nuclear staining. C: cytoplasmic staining. * localized intra-nuclear reactions. Abbreviations: Spg: spermatogonia. Spc: spermatocytes. rSpt: round spermatids. eSpt: elongated spermatids. Ser: Sertoli cells. Ley: Leydig cells. PMC: peritubular myoid cells of the seminiferous tubules. Antibody designations are specified in Table S4. Related to Figure 3.

Supplementary Table 2

Gene	Fwd Primer 5'-3'	Rev Primer 5'-3'	Amplicon size	GenBank Accesion no.
Rankl (Tnfsf11)	AGCCGAGACTACGGCAAGTA	AAAGTACAGGAACAGAGCGATG	201 bp	NM_011613.3
Rank (Tnfrsf11a)	GGACGGTGTTGCAGCAGAT	GCAGTCTGAGTTCCAGTGGTA	243 bp	NM_009399.3
Opg (Tnfrsf11b)	ACCCAGAAACTGGTCATCAGC	CTGCAATACACACACTCATCACT	157 bp	NM_008764.3
Nfkb1	ATGGCAGACGATGATCCCTAC	CGGAATCGAAATCCCCTCTGTT	167 bp	NM_008689.2
Nfkb2	TGGCATCCCCGAATATGATGA	TGACAGTAGGATAGGTCTTCCG	212 bp	NM_019408.3
Inhibin β-B (Inhbb)	GCCCAACATGATTGTGGAGGAGTG	CATTTGTCACCGCATCCATTTG	319 bp	NM_008381.3
B2m	CGAGACATGTGATCAAGCATCA	TATTGCTCAGCTATCTAGGATAT	199 bp	NM_009735.3
Amh	GCGCGGGGACAAGGGACA	CAGCCGCACTCGGTGGCTA	325 bp	NM_007445

Supplementary Table 2. Primer sequences used for gene expression analyses in mice. Related to Figure 1-2 and Materials and Methods.

Supplementary Table 3

Gene	Forward primer 5’-3’	Reverse primer 5’-3	Amplicon size	GenBank Accesion no.
RANKL (*TNFSF11)*	TCGTTGGATCACAGCACATCA	TATGGGAACCAGATGGGATGTC	141 bp	NM_003701_3
RANKL (TNFSF11)	TGGATCACAGCACATCAGAGCAG	TGGGGCTCAATCTATATCTCGAAC	556 bp	NM_003701.4
RANK (*TNFRSF11A*)	AGATCGCTCCTCCATGTACCA	GCCTTGCCTGTATCACAAACTTT	205 bp	NM_003839.4
OPG (*TNFRSF11B*)	CACAAATTGCAGTGTCTTTGGTC	TCTGCGTTTACTTTGGTGCCA	216 bp	NM_002546.4
B2M	ATCCAATCCAAATGCGGCATC	AGTATGCCTGCCGTGTGAAC	113	NM_004048.3
RPS20	AGACTTTGAGAATCACTACAAGA	ATCTGCAATGGTGACTTCCAC	179 bp	NM_001023.3

Supplementary Table 3. Primer sequences used for gene expression analyses of human testis specimens. Different primer sets were used for RT-PCR and qRT-PCR analysis. Related to Figure 3 and Materials and Methods.

Supplementary Table 4

Antibody designation	Species	Target	Provider (cat. no.)
RANKL C-term	Rabbit	Human soluble receptor activator of NF-Kappa B Ligand	Abcam (Ab-9957)
RANKL TM	Rabbit	Amino acids 46-317 of full length RANKL of human origin	Santa Cruz Biotech (sc-9073)
RANKL N-term	Goat	Peptide mapping at the N-terminus of mouse RANKL	Santa Cruz Biotech (sc-7628)
RANKL extrac.	Mouse, monoclonal	Amino acids 74-308 of human RANKL	Novus biologicals (NBP2-61813, clone 8A7B9)
RANK C-term	Rabbit	Amino acids 317-616 of human RANK	Santa Cruz Biotech (sc-9072)
RANK Internal	Rabbit	Amino Acids 261-379 of RANK	Atlas Antibodies (HPA027728)
OPG N-term	Goat	Peptide mapping near the N-terminus of human OPG	Santa Cruz Biotech (sc-8468)
OPG N-term 2	Goat	Peptide mapping near the N-terminus of human OPG	Santa Cruz Biotech (sc-21038)
D2-40	Mouse, monoclonal	Human Podoplanin, Clone D2-40	Dako (M3619)
cPARP	Rabbit, monoclonal	Large fragment of human PARP1 protein produced by caspase cleavage	Cell Signaling Technology (5625)
BrdU	Mouse, monoclonal	Bromodeoxyuridine	Dako (M0744)
GATA-4	Goat	C-terminus of mouse GATA-4	Santa Cruz Biotech (sc-1237)
OCT-3/4	Mouse, monoclonal	Amino acids 1-134 of human OCT-3/4	Santa Cruz Biotech (sc-5279)
MAGE-A4	Mouse, monoclonal	Mage-A4	Gift from Giulio Spagnoli, Clone 57B, 4C
SOX9	Rabbit	SOX9	Millipore (AB5535)
VASA/DDX4	Rabbit	Human DDX4/ MVH aa 700 to the C-terminus (C terminal)	Abcam, (Ab-13840)
β-actin	Mouse, monoclonal	Gizzard Actin of avian origin	Santa Cruz Biotech (sc-47778)

Supplementary Table 4. Primary antibodies used in the study and their protein targets. The antibodies used for western blotting, immunohistochemistry and immunofluorescence. The specificity of the antibodies was tested in bone and breast tissue. Abbreviations: Cat. no., catalogue number. Related to Figure 1-4.

Supplementary Table 5

Antibody designation	Dilution (WB)	Retrieval buffer (IHC std./ IHC P.C.)	Dilution (IHC std. / IHC P.C.)	Dilution (IF, Human/Mouse)	Retrieval buffer (IF)
RANKL C-term	1:200	CIT / TEG	1:150 / 1:1100	-	-
RANKL TM	1:200	CIT / TEG	1:250* / 1:250	1:350	TEG
RANKL N-term	-	CIT / TEG	1:200 / 1:500	1:500	TEG
RANKL extrac.	1:200	TEG / TEG	- / 1:10.000	-	-
RANK C-term	1:200	CIT / CIT	1:100 / 1:600	-	-
RANK Internal		TEG / TEG	1:200 / 1:450	1:450	TEG
OPG N-term	1:200	CIT / TEG	1:100 / 1:600	-	-
OPG N-term 2	-	CIT / TEG	1:100 / 1:400	1:400	TEG
D2-40	-	CIT / TEG	1:100 / 1:10.000	-	-
cPARP	-	CIT / -	1:100 / -	-	-
BrdU	-	CIT / -	1:100 / -	-	-
GATA-4	-	TEG / -	1:100 / -	-	-
OCT-3/4	-	TEG / -	1:100 / -	-	-
MAGE-A4	-	CIT / TEG	1:500 / 1:3000	1:1000/-	TEG
SOX9	-	-	-	1:8000/1:3000	CIT
VASA/DDX4	-	-	-	-/1:5000	TEG
β-actin	1:200	-	-	-	-
**Secondary antibody**	**Species**	**Provider (cat. no.)**	**Dilution (WB/IF)**	**IHC std.**	**IHC P.C.**
Anti-rabbit	Swine	Dako (P0217)	1:1000/-	-	-
Anti-mouse	Rabbit	Dako (P0260)	1:1000/-	-	-
Anti-goat	Rabbit	Dako (P0160)	1:1000/-	-	-
Anti-rabbit	Horse	Vector (MP-7401)	-/-	-	X
Anti-goat	Horse	Vector (MP-7401)	-/-	-	X
Anti-mouse	Horse	Vector (MP-7402)	-/-	-	X
Antimouse/ rabbit	Goat	Zymed Laboratories (956543)	-/-	X	-
Anti-goat	Donkey	Binding Site (ab260CUS01)	-/-	X	-
Anti-mouse peroxidase	Chicken	Santa Cruz (sc-2962)	-/1:200	-	-
Anti-rabbit	Chicken	Santa Cruz (sc-2963)	-/1:200	-	-
Anti-goat	Chicken	Santa Cruz (sc-2961)	-/1:200	-	-

Supplementary table 5. Antibody dilutions, retrieval buffer, details, and information regarding secondary antibodies. For IHC, antigen retrieval was conducted by microwaving or placing sections in a pressure cooker in indicated retrieval buffer. Citrate buffer: 10 mM, pH 6.0; TEG buffer: 10 mM Tris, 0.5 mM EGTA, pH 9.0. * For staining of spermatozoa antibody sc-9073 was used with TEG buffer at 1:100. Abbreviations: Cat. No., catalogue number; IF, immunofluorescence; IHC, immunohistochemistry; P.C., Pressure Cooker, WB, western blot; X, indicates the application for the secondary antibody. Related to Figure 1-4.

Supplementary Table 6

Variable	Healthy men			Infertile men		
	Mean	SD	N	Mean	SD	N
Age (years)	19.41	1.59	153	34.76	6.57	330
BMI (kg/m^2)	22.26	2.78	150	26.51	4.20	317
sRANKL serum* (pmol/L)	0.35	1.72	151	0.15	2.21	302
sRANKL seminal fluid* (pmol/L)	13.82	2.50	152	23.81	2.16	217
LH (IU/L)	3.04	1.84	153	4.43	2.44	301
FSH (IU/L)	2.95	1.46	153	5.30	4.15	301
Testosterone (nmol/L)	20.17	27.95	153	14.40	4.60	301
Estradiol (pmol/L)	56.46	64.86	153	100.32	26.83	301
SHBG (nmol/L)	31.53	4.58	153	30.68	12.08	301
Inhibin B (pg/mL)	186.67	10.20	153	158.56	78.08	301
Inhibin B/FSH-ratio	93.45	70.57	153	55.26	65.03	301
Testicular size (mL)	13.74	1.32	153	12.71	4.20	275
Semen volume (mL)	3.23	172.16	153	3.85	1.79	323
Total sperm count* (10^6)	110.94	3.04	153	31.99	7.18	323
Sperm concentration* (10^6/mL)	37.17	3	153	8.95	7.14	330
Progressive sperm motility (%)	65.57	4.72	153	31.86	19.80	315
Sperm morphology (%)	7.67	3.80	149	3.32	2.93	320

Baseline characteristics table showing hormones, seminal parameters, and testicular size of healthy and infertile men included in analyses of RANKL in seminal fluid.

** transformed with natural logarithm to obtain Gaussian distribution, values back-transformed in table*

Supplementary table 6. Baseline characteristics of healthy and infertile men included in analyses of RANKL in serum and seminal fluid. Table showing hormones, seminal parameters, and testicular size of all men included in the analyses of soluble RANKL in seminal fluid and serum. All data presented as mean and SD. BMI, Body mass index; sRANKL, soluble RANKL; LH, luteinizing hormone; AMH, Anti-Müllerian hormone; FSH, Follicle-stimulating hormone; SHBG, Sex hormone-binding globulin. Related to Figure 5.

Supplementary Table 7

Investigated parameter	Beta-value	P-value
Volume (mL)	NA	NS
Total sperm* (10^6)	NA	NS
Sperm concentration* (10^6/mL)	-0.001	0.009
Progressive sperm motility (%)	-0.019	<0.0001
Sperm motility (%)	-0.017	<0.0001
Sperm morphology (%)	-0.002	0.003
Number of progressive motile sperm *	-0.001	0.0001
Number of morphological normal sperm *	-0.001	0.001

Table showing linear regression of semen quality and RANKL-seminal/serum-ratio.
** transformed with natural logarithm to obtain Gaussian distribution.*

Supplementary Table 7. Linear regression analysis of semen quality and seminal fluid/serum RANKL ratio. Pooled analyses of the seminal fluid/serum RANKL ratio of all the men. All hormonal analyses were adjusted for BMI and analyses on semen quality were further adjusted for time of abstinence. * transformed with natural logarithm to obtain Gaussian distribution. Abbreviations: NA, not applicable; NS, not significant. Related to Figure 5.

Supplementary Table 8

Variable	
N	12
BMI (kg/m^2)	26.3 (3.3)
Total sperm count (10^6)	24 (15)
Sperm concentration (10^6/mL)	8.8 (7.3)
Sperm motility (ABC) (%)	30 (15)
Progressive sperm motility (%)	24 (15)
Inhibin B (pg/mL)	170 (87)
AMH (pmol/L)	48 (21)
FSH (U/L)	4.6 (3.1)
Testosterone (nmol/L)	15.4 (4.9)
Estradiol (pmol/L)	55.5 (14.2)
SHBG (nmol/L)	36.1 (9.1)
Calcium (mmol/L)	2.37 (0.04)
Phosphate (mmol/L)	0.96 (0.13)
PTH (pmol/L)	3.3 (0.7)
1,25(OH)$_2$D$_3$ (pmol/L)	77 (21)
25(OH)D > 50 nmol	12

Baseline characteristics of all included men in the intervention study with Denosumab study. All data presented as mean. Median total sperm count was 22 million and median sperm concentration was 7.6 million/ml

Supplementary Table 8. Baseline characteristics of 12 infertile men included in intervention study with Denosumab. Baseline characteristics of all included men in the intervention study with Denosumab study. All data presented as mean and SD. Median total sperm count was 22 million and median sperm concentration was 7.6 million/ml. Abbreviations: BMI, Body mass index; AMH, Anti-Müllerian hormone; FSH, Follicle-stimulating hormone; SHBG, Sex hormone-binding globulin; PTH, Parathyroid hormone. Related to Figure 6.

Supplementary Table 9

Hormone	Platform	Producer	Cat. No.	CV
LH	Time-resolved immuno-fluorometric assay	Delfia; Wallac, Turku, Finland		6%
FSH	Time-resolved immuno-fluorometric assay	Delfia; Wallac, Turku, Finland		4%
Inhibin B	Two-sided enzyme linked immunoassay	Inhibin B genII, Beckman Coulter, USA	A81301	11%
AMH	Enzyme immunometric assay	Immunotech, Beckman Coulter	A79765	8%
Estradiol	Access RIA	Pantex, Santa Monica, USA		13%
Testosterone	Access RIA	Coat-a-Count, Siemens	TKTT2	6%
sRANKL	ELISA	Biomedica Austria	BI-20462	3%
Total RANKL	ELISA	Immundiagnostik, Austria	K 1016	12%
OPG	ELISA	Biomedica, Austria	BI-20403	5%
Klotho	ELISA	ibl-international, Switzerland	JP27998	8%
25-OH vitamin D	LC-MS	Holbæk Hospital, Denmark	-	9%
1,25-OH_2 vitamin D	LC-MS	Aarhus University, Denmark	-	18%

Supplementary Table 9. Biochemical analyses used in the study. Related to figure 5-6 and Materials and Methods.

Supplementary Table 10

A number of animal facility reports showing how the predominant lactation Deficit in RANKl -/- females causes a high proportion of dead pups. Table showing lactation deficit data recorded for SC-*Rankl* (Amh-cre) and *Rankl* -/- (Vasa-cre) mice. Data from Study no. 211-002 Breeding cages F 2. Related to figure 2.

	Born (week/year)	Pups	Weaned	Female cage no.	Male cage no.	Remark
Breeding Cage no. 11	08/2015	5	11/2015	3	1	1 eat
Start week: 04/2015	15/2015	8	19/2015	2	6	-
Male: Vasa-cre no. 5	23/2015	10	27/2015	3	4	3 dead
Female Rankl	30/2015	2	34/2015	2	-	-
Breeding cage no. 12	07/2015	3	-	-	-	Dead
Start week: 04/2015	10/2015	8	14/2015	2	2	4 dead
Male: Rankl	17/2015	1	-	-	-	1 eaten
Female: Vasa-cre no. 4	22/2015	4	26/2015	1	-	1 eaten<br>2 dead
	30/2015	4	-	-	-	3 unwell<br>1 dead
	33/2015	1	-	-	-	1 dead
	44/2015	1	-	-	-	1 dead
Breeding cage no. 13	09/2015	1	-	-	1	7 stillborn
Start week: 04/2015	12/2015	5	17/2015	-	1	4 eaten
Male: Rankl	15/2015	3	-	-	-	Dead
Female: Vasa-cre no. 16	19/2015	4	22/2015	2	1	1 dead
	21/2015	2	-	-	-	2 dead
	24/2015	3	-	-	-	3 dead
	27/2015	5	31/2015	2	-	1 dead<br>2 eaten
	33/2015	7	37/2015	2	1	2 dead<br>2 unwell
	36/2015	1	-	-	-	1 dead
	46/2015	3	-	-	-	3 dead
Breeding cage no. 14	07/2015	7	11/2015	1	1	5 eaten
Start week: 04/2015	10/2015	9	14/2015	-	1	8 eaten
Male: Rankl	13/2015	8	17/2015	3	2	3 eaten
Female: Vasa-cre no. 17	19/2015	4	23/2015	-	2	2 dead
	22/2015	7	26/2015	2	3	2 dead
Breeding cage no. 15	08/2015	7	11/2015	3	2	2 eaten
Start week: 04/2015<br>Male: Vasa-cre no. 20	17/2015	10	20/2015	3	3	2 eaten<br>2 dead
Female: Rankl	20/2015	3 ½	Dead born			
	23/2015	8	26/2015	3	4	1 dead
	27/2015	2	31/2015	-	2	-
	38/2015	1	-	-	-	1 eaten
Breeding cage no. 16	10/2015	6	13/2015	1	5	-
Start week: 04/2015	16/2015	6	19/2015	4	1	1 dead
Male: Rankl	19/2015	1	-	-	-	Unwell
Female: Vasa-cre no. 24	22/2015	5	25/2015	3	2	-
	25/2015	1	-	-	-	1 dead
Breeding cage no. 17	08/2015	3	11/2015	1	2	-
Start week: 04/2015	11/2015	2	15/2015	1	1	-
Male: Vasa-cre no. 25	14/2015	6	18/2015	1	3	2 dead
Female: Rankl	17/2015	2	21/2015	1	-	1 dead

	20/2015	2	24/2015	2	-	-
	23/2015	2	26/2015	2	-	-
	26/2015	2	-	-	-	-
	30/2015	4	33/2015	1	2	1 unwell
	33/2015	1	-	-	-	1 dead
	36/2015	6	40/2015	4	2	-
Breding cage no. 18<br>Start week: 04/2015<br>Male: Rankl<br>Female: Vasa-cre no. 45	07/2015	5	11/2015	2	-	3 eaten
	10/2015	4	142015	-	2	2 dead
	14/2015	3	17/2015	-	3	-
	20/2015	9	23/2015	4	3	2 dead
	26/2015	3	29/2015	-	2	1 dead
	29/2015	2	-	-	-	1 eaten<br>1 dead
	33/3015	5	36/2015	2	2	1 dead
	42/2015	1	-	-	-	1 dead
Breeding cage no. 19<br>Start week: 04/2015<br>Male: Vasa-cre no. 48<br>Female: Rankl	08/2015	7	11/2015	4	3	-
	11/2015	5	15/2015	2	3	-
	15/2015	3	19/2015	-	2	1 dead
	18/2015	4	21/2015	1	3	-
	27/2015	4	31/2015	2	1	1 dead
	39/2015	2	43/2015	1	-	1 dead
Breeding cage no. 20<br>Start week: 04/2015<br>Male: Vasa-cre no. 50<br>Female: Rankl	08/2015	6	11/2015	2	3	1 dead
	13/2015	5	17/2015	2	3	4 dead
	22/2015	7	25/2015	3	2	2 unwell,<br>teeth
	28/2015	5	31/2015	5	-	-
	31/2015	2	-	-	-	Stillborn
	36/2015	6	40/2015	1	5	-
Breeding cage no. 21<br>Start week: 04/2015<br>Male: Rankl<br>Female: Amh-cre no. 7	08/2015	7	11/2015	5	2	-
	11/2015	4	14/2015	2	2	-
	17/2015	11	17/2015	5	6	-
	21/2015	8	24/2015	1	7	-
	27/2015	11	30/2015	4	7	--
	31/2015	4	34/2015	4	-	-
	34/2015	4	37/2015	1	3	-
	41/2015	2	44/2015	-	2	-
	44/2015	3	47/2015	3	-	-
	47/2015	1	-	-	-	Eaten
Breeding cage no. 22<br>Start week: 04/2015<br>Male: Rankl<br>Female: Amh-cre no. 11	08/2015	5	11/2015	2	3	-
	13/2015	9	16/2015	5	4	-
	17/2015	3	19/2015	2	1	-
	19/2015	1	-	-	-	1 dead
	25/2015	9	28/2015	8	1	-
	28/2015	1	-	-	-	1 dead
	32/2015	10	36/2015	6	3	1 eaten
	39/2015	6	42/2015	4	2	-
	42/2015	2	-	-	-	Stillborn
	43/2015	2	48/2015	2	-	-
	49/2015	8	52/2015	5	3	-
Breeding cage no. 23<br>Start week: 04/2015<br>Male: Rankl	07/2015	9	10/2015	4	5	-
	11/2015	5	14/2015	2	3	-
	14/2015	7	17/2015	4	2	1 eaten

Female: Amh-cre no. 38	17/2015	6	20/2015	4	2	-
	20/2015	2	25/2015	2	-	-
	25/2015	11	28/2015	5	6	-
	29/2015	3	32/2015	1	-	1 dead<br>1 unwell
	32/2015	2	35/2015	-	2	-
	35/2015	5	38/2015	3	2	-
	38/2015	1	-	-	-	1 dead
Breeding cage no. 24<br>Start week: 04/2015<br>Male: Rankl<br>Female: Amh-cre no. 39	07/2015	7	10/2015	2	5	-
	11/2015	9	15/2015	2	6	1 eaten
	17/2015	8	20/2015	4	4	-
	25/2015	8	28/2015	5	3	-
	33/2015	10	36/2015	6	4	-
	41/2015	7	44/2015	2	5	-
	45/2015	6	48/2015	-	6	-
Breeding cage no. 25<br>Start week: 04/2015<br>Male: Rankl<br>Female: Amh-cre no.25	07/2015	5	10/2015	3	2	-
	13/2015	10	16/2015	7	3	-
	16/2015	10	19/2015	2	6	2 eaten
	22/2015	11	26/2015	4	7	-
	29/2015	10	32/2015	6	4	-
	32/2015	9	35/2015	6	3	-
	39/2015	7	42/2015	1	4	1 dead<br>1 eaten
	42/2015	1	-	-	-	1 dead
	53/2015	4	03/2016	3	1	-
	08/2016	4	-	1	3	-
Breeding cage no. 26<br>Start week: 04/2015<br>Male: Amh-cre no. 13<br>Female: Rankl	08/2015	4	11/2015	2	2	-
	14/2015	7	18/2015	4	3	-
	19/2015	6	23/2015	1	5	-
	24/2015	4	-	-	-	-
Breeding cage no. 27<br>Start week: 04/2015<br>Male: Amh-cre no. 35<br>Female: Rankl	07/2015	6	11/2015	3	3	-
	11/2015	2	15/2015	2	-	-
	15/2015	5	18/2015	1	4	-
	20/2015	4	24/2015	2	2	-
	24/2015	1	-	-	-	1 dead
	27/2015	3	-	-	-	1 dead<br>2 eaten
	34/2015	1	-	-	-	1 unwell
Breeding cage no. 28<br>Start week: 04/2015<br>Male: Amh-cre no. 36<br>Female: Rankl	07/2015	8	11/2015	6	2	-
	11/2015	7	14/2015	4	3	-
	24/2015	4	-	-	-	-
	Female unwell					
Breeding cage no. 29<br>Start week: 04/2015<br>Male: Amh-cre no. 37<br>Female: Rankl	08/2015	8	11/2015	5	3	-
	12/2015	10	15/2015	5	5	-
	17/2015	6	20/2015	3	2	1 dead
	20/2015	6	23/2015	4	2	-
	24/2015	4	27/2015	2	2	-
	Female unwell					
Breeding cage no. 30<br>Start week: 04/2015<br>Male: Amh-cre no. 42<br>Female: Rankl	07/2015	3	11/2015	3	-	-
	13/2015	9	17/2015	3	6	-
	Female unwell					

Supplementary Table 11

Supplementary Table S11 A number of animal facility reports that failure of Tooth eruption was a relatively requent finding in mice with global RANKL deficiency. Data from study no. 211-002 Breeding cages F 4. Related to figure 2.

	Born (week/year)	Pups	Weaned	Female cage no.	Male cage no.	Remark
Breeding Cage no. 52	46/2015	4	50/2015	3	1	-
Start week: 43/2015<br>Male F3: no. 511	49/2015	2	-	-	-	1 eaten<br>1 dead
Female F3: no. 504	53/2015	7	03/2015	4	1	2 eaten<br>2 - teeth
	03/2016	1	06/2016	-	1	-
	06/2016	2	-	1	1	-
Breeding Cage no. 53<br>Start week: 43/2015	46/2015	9	50/2015	1	4	3 eaten<br>1 unwell
Male F3: no. 507	50/2015	4	1/2016	2	2	-
Female F3: no. 516	53/2015	1	03/2016	-	1	-
	03/2016	9	06/2016	2	7	-
	07/2016	5	10/2016	2	1	2 eaten
	13/2016	8				
Breeding Cage no. 54	46/2016	8	50/2015	3	3	2 eaten
Start week: 43/2015	53/2015	9	03/2016	5	3	1 unwell
Male F3: no. 513	06/2016	7	09/2016	6	1	-
Female F2: no. 602	10/2016	10	13/2016	6	-	3 unwell
	13/2016	7				
Breeding Cage no. 55	47/2015	5	50/2015	3	-	2 dead
Start week: 43/2015	50/2015	8	01/2016	3	3	2 - teeth
Male F3: no. 536	01/2016	7	04/2016	2	4	1 unwell
Female F2: no. 603	04/2016	4	-	-	-	4 dead
	08/2016	9	4/2016	3	5	1 dead
	11/2016	3	-	-	-	1 dead<br>2 eaten
Breeding Cage no. 56	46/2015	8	50/2016	2	3	3 unwell
Start week: 43/2015	50/2015	8	01/2016	2	1	5 - teeth
Male F3: no. 539	02/2016	11	05/2016	6	5	-
Female F2: no. 604	08/2016	11	12/2016	4	5	2 dead
	12/2016	8				
Breeding Cage no. 57	47/2015	7	50/2015	3	4	-
Start week: 43/2015	51/2015	8	01/2016	4	4	-
Male F3: KO no. 560	01/2016	2	-	-	-	stillborn
Female F2: no. 605	05/2016	7	07/2016	3	4	-
	08/2016	5	11/2016	3	2	-
	11/2016	4	2 dead, 1<br>eat	11/2016	4	2 dead<br>1 eaten
Breeding Cage no. 58	47/2015	1	-	-	-	1 eaten
Start week: 43/2015	50/2015	5	01/2016	-	5	-
Male F3: no. 568	01/2016	4	04/2016	3	1	-
Female F2: no. 607	06/2016	6	08/2016	3	3	-
	09/2016	2	12/2016	2	-	-
	12/2016	2	1 eat	12/2016	2	1 eaten
Breeding Cage no. 59	50/2015	8	01/2016	2	4	2 dead
Start week: 43/2015	03/2016	6	06/2016	4	2	3 dead

Male F3: no. 569<br>Female F2: no. 614	08/2016	9	12/2016	6	1	2 dead
	13/2016	5				
Breeding Cage no. 60<br>Start week: 43/2015<br>Male F3: no. 571<br>Female F2: no. 616	47/2015	5	50/2016	3	1	1 dead
	50/2015	5	01/2016	5	-	-
	01/2016	5	03/2016	-	5	-
	04/2016	6	07/2016	2	3	1 dead
	07/2016	2	-	-	-	stillborn
	10/2016	7	13/2016	4	3	-

Study no. 211-002 Breeding cages Control

	Born (week/year)	Female	Pups	Weaned	Female cage no.	Male cage no.	Remark
Breeding Cage no. 67<br>Start week: 51/2015<br>Male: no. 640<br>Female: no. 750 (WT f/f), 742 (Wt f/f), 735 (Amh KO)	02/2016	735	2	-	-	-	Stillborn
	03/2016	742	7	06/2016	1	4	2 unwell teeth
	02/2016	750	-	-	-	-	Stillborn pups
	05/2016	735	7	08/2016	1	3	2 dead<br>1 - teeth
	07/2016	750	10	10/2016	4	3	3 unwell
	09/2016	742	9	14/2016	5	2	2 unwell
	12/2016	735	6				
	13/2016	750	14				
Breeding Cage no. 68<br>Start week: 51/2015<br>Male: no. 697<br>Female: no. 732, 700, 587	03/2016	732	9	06/2016	5	2	2 unwell - teeth
	02/2016	700+587	20	06/2016	5	11	4 unwell - teeth
	10/2016	732+587	14	13/2016	5	8	1 eat
	10/2016	700	7				
	13/2016	587	3				

Supplementary References

1. Van Cromphaut,S.J. *et al.* Duodenal calcium absorption in vitamin D receptor-knockout mice: functional and molecular aspects. *Proc. Natl. Acad. Sci. U. S. A* **98**, 13324-13329 (2001).
2. Blomberg,Jensen .M. *et al.* Characterization of the testicular, epididymal and endocrine phenotypes in the Leuven Vdr-deficient mouse model: Targeting estrogen signalling. *Mol. Cell Endocrinol.* **377**, 93-102 (2013).
3. E. Rotgers *et al.*, Retinoblastoma protein (RB) interacts with E2F3 to control terminal differentiation of Sertoli cells. *Cell Death. Dis.* 5, e1274 (2014).
