# Supplementary material for: RANKL regulates male reproductive function
Source: Nat Commun. 2021 Apr 23;12:2450. doi: 10.1038/s41467-021-22734-8 (PMC8065035; doi:10.1038/s41467-021-22734-8)
Supplement: Supplementary file 3 — Reporting Summary [file 41467_2021_22734_MOESM3_ESM.pdf]

nature research

Corresponding author(s): Martin Blomberg Jensen
Last updated by author(s): Mar 15, 2021

Reporting Summary

Nature Research wishes to improve the reproducibility of the work that we publish. This form provides structure for consistency and transparency in reporting. For further information on Nature Research policies, see Authors & Referees and the Editorial Policy Checklist.

Statistics

For all statistical analyses, confirm that the following items are present in the figure legend, table legend, main text, or Methods section.

n/a	Confirmed	
☐	☒	The exact sample size (*n*) for each experimental group/condition, given as a discrete number and unit of measurement
☐	☒	A statement on whether measurements were taken from distinct samples or whether the same sample was measured repeatedly
☐	☒	The statistical test(s) used AND whether they are one- or two-sided<br>*Only common tests should be described solely by name; describe more complex techniques in the Methods section.*
☐	☒	A description of all covariates tested
☐	☒	A description of any assumptions or corrections, such as tests of normality and adjustment for multiple comparisons
☐	☒	A full description of the statistical parameters including central tendency (e.g. means) or other basic estimates (e.g. regression coefficient) AND variation (e.g. standard deviation) or associated estimates of uncertainty (e.g. confidence intervals)
☐	☒	For null hypothesis testing, the test statistic (e.g. F, t, r) with confidence intervals, effect sizes, degrees of freedom and P value noted<br>*Give P values as exact values whenever suitable.*
☐	☒	For Bayesian analysis, information on the choice of priors and Markov chain Monte Carlo settings
☐	☒	For hierarchical and complex designs, identification of the appropriate level for tests and full reporting of outcomes
☐	☒	Estimates of effect sizes (e.g. Cohen's *d*, Pearson's *r*), indicating how they were calculated

Our web collection on statistics for biologists contains articles on many of the points above.

Software and code

Policy information about availability of computer code

Data collection	data were not collected using a specific system or software.
Data analysis	IBM SPSS statistics version 25, Morphometric analysis was performed following scanning of sections on a NanoZoomer 2.0 HT (Hamamatsu Photonics) and images captured using the software NDPview version 2.6.13 (Hamamatsu Photonics).

For manuscripts utilizing custom algorithms or software that are central to the research but not yet described in published literature, software must be made available to editors/reviewers. We strongly encourage code deposition in a community repository (e.g. GitHub). See the Nature Research guidelines for submitting code & software for further information.

Data

Policy information about availability of data

All manuscripts must include a data availability statement. This statement should provide the following information, where applicable:

- Accession codes, unique identifiers, or web links for publicly available datasets
- A list of figures that have associated raw data
- A description of any restrictions on data availability

All data are available from the authors

Field-specific reporting

Please select the one below that is the best fit for your research. If you are not sure, read the appropriate sections before making your selection.

☒ Life sciences ☐ Behavioural & social sciences ☐ Ecological, evolutionary & environmental sciences

Life sciences study design

All studies must disclose on these points even when the disclosure is negative.

Sample size	Sample size was determined using power estimate. human studies were approved by ethical comitte and they require power estimate prior to approval. sample size in the rodent models was determined by using the efficacy study in wildtype mice with OPG treatment
Data exclusions	All mice were included after the genotype was validated by PCR and tomato expression and none of the mice were excluded. In the human intervention study one man was excluded because he had one of the predefined exclusion criteria (high fever) as stated in the manuscript.
Replication	Treatment with OPG in wildtype mice were two independent identical experiments in wildtype mice. WB were replicated at least once, while IHC and IF were conducted on minimum three different genotypes, tissue types with reproducible data to be used in the manuscript. Tissues with malignant or tissue with poor Morphology were discarded and not used for IHC or IF.<br>PCR were run in triplicates.<br>Testicular tissue used for ex vivo cultures were discarded if histology was poor or evidence of starvation prior to any analyses . Functional test on human spermatozoa were conducted on three sperm donors
Randomization	No randomization was conducted but all the technicians handling the animals and conducting the fertility study was blinded to the genotype.In all the human studies, initially the unadjusted data were reported and then we report the data after adjustment of relevant confounders. The confounders used depend on the outcome-variable and the availability of the data. This is highlighted in the statistical section and in the results section.
Blinding	All investigators and technicians were blinded during data handling and analysis.

Reporting for specific materials, systems and methods

We require information from authors about some types of materials, experimental systems and methods used in many studies. Here, indicate whether each material, system or method listed is relevant to your study. If you are not sure if a list item applies to your research, read the appropriate section before selecting a response.

Materials & experimental systems

n/a	Involved in the study
☐	☒ Antibodies
☒	☐ Eukaryotic cell lines
☒	☐ Palaeontology
☐	☒ Animals and other organisms
☐	☒ Human research participants
☐	☒ Clinical data

Methods

n/a	Involved in the study
☒	☐ ChIP-seq
☒	☐ Flow cytometry
☒	☐ MRI-based neuroimaging

Antibodies

Antibodies used	They have all been listed in a Table in the Supplementary material with all relevant information
Validation	All the primary antibodies have been validated in at least on positive control tissue to test whether the observed expression was in accordance with existing data<br><br>RANKL C-term<br><br>RANKL TM<br><br>RANKL N-term<br><br>RANKL extrac.<br><br>RANK C-term<br><br>RANK Internal<br><br>OPG N-term<br><br>OPG N-term 2<br><br>Were all tested on human breast tissue and/or bone

cPARP

BrdU

Were validated on different tissue cultures using specific induceres of apoptosis or proliferation

D2-40

OCT-3/4

SOX9
validated on germ cell neoplasia in situ and/or invasive testicular germ cells tumours

GATA-4

VASA/DDX4

MAGE-A4
human and/or mouse testis.

Animals and other organisms

Policy information about studies involving animals; ARRIVE guidelines recommended for reporting animal research

Laboratory animals

Rankl floxed mice (Ranklfl/fl), C57BL/6 (WT male and female) and VasaCre mice were purchased from Jax Mice/Javier Mice, and MisCre (Amh:Cre) mice were provided by Jorma Toppari (Turku, Finland). Detailed breeding details can be found in supplementary material . Male mice were used and sacrificed 15-17 weeks of age or 10 weeks for wt mice
The mice were caged in European standard cages type II. Bedding will be Jeluxyl HW 300/500 (Jelu, J.Ehrler GmbH & Co KG, Ludwigsmühle, D-73494 Rosenberg, Germany). There was a hide, nesting material (soft paper wool (Cat. No. 1034007), LBS Biotechnology, Surrey, UK) and a biting stick in the cage. The bedding was changed once a week in a laminar flow unit. The air was exchanged approximately 12 times per hour in the stable. Temperature was between 20C to 24C and was controlled via the ambient ventilation system. Light cycle was 12-hour dark and 12-hour light (lights on 06.00).

Diet and Water
Diet was Altromin 1324, produced by Altromin, Im Seelenkamp 20, 32791 Lage, Germany. Water was UV-sterilized and water bottles were refilled when necessary during acclimatization and experiment. Diet and water were administered ad libitum.

Clinical observations
All animals are inspected on a daily basis for their general condition. Any clinical signs or behavioural abnormalities were recorded. The clinical observations are described in results section.

Humane end points and premature termination
Any animal showing clinical signs of moderate pain or moderate distress or any degree of suffering would have been humanely euthanized. Further would any animal have been humanely euthanized if it is exhibiting clinical signs that exceed the limits of the study specific humane end points according to the European and Danish legislation on animals in experimental studies.

The animals did have (murine pathogene free) MPF-status and the housing and changing system is designed to assure that the MPF-status was preserved during the study. Educated personnel under veterinary supervision were handling the animals.

Wild animals

No wild animals were used

Field-collected samples

No field collected samples were used

Ethics oversight

. All animal studies were approved by the Danish Animal Experiments Inspectorate (license number 2011/561-2006). Animals were housed and cared for according to National ethical guidelines

Note that full information on the approval of the study protocol must also be provided in the manuscript.

Human research participants

Policy information about studies involving human research participants

Population characteristics

Copenhagen Bone Gonadal Study NCT01304927: A double blind randomized clinical trial in which 307 infertile men were included and subsequently supplemented with either cholecalciferol 300.000 IU daily followed by 1400 IU + 500 mg calcium daily or placebo for 5 months. The study was approved by the regional ethics committee and EMA approval no. 2010-024588-42, H-4-2010-138, and 2010124801. All men were part of an infertile couple and had impaired semen quality and delivered two semen samples, underwent physical investigation including ultrasound of genitals, full body, columnar and hip DXA scan, delivered fasting serum samples prior to the intervention. Only collected data prior to the intervention here. More details can be found in Supplementary Table 6 and at https://clinicaltrials.gov/ct2/show/NCT01304927?term=blomberg+jensen&rank=4.

Human tissue samples and sperm function tests: Patients were recruited from Department of Growth and Reproduction, Rigshospitalet, Denmark in accordance with the Helsinki Declaration after approval from the local ethics committee

(H-17004362). Adult testis tissue was obtained from orchidectomy specimens performed due to testicular cancer and specimens with invasive cancer were discarded after evaluation of IHC staining with PLAP, OCT4, or D2-40 antibodies. The non-malignant tissue was used for these studies as described in detail in Supplementary Methods 4. Healthy sperm donors were used to study the effects on Denosumab on human spermatozoa.

TNFSF11 Inhibition and Fertility: a Prospective intervention Study NCT02422108: To address the relevance of RANKL inhibition on human male reproduction, Denosumab (Prolia) 60 mg was injected into 12 infertile men once and semen quality was monitored for 180 days (https://clinicaltrials.gov/ct2/show/NCT02422108). The study was approved by the local ethics committee (H-15001992) and was conducted and monitored according to GCP standard after informed consent was obtained from all participants. All 12 infertile men had impaired semen quality and the severity varied from mild to severe oligospermia (Supplementary Table 8). Their bone health evaluated by DXA showed normal BMD and all had normal serum calcium, phosphate, alkaline phosphatase, 25-OHD and PTH levels at baseline. All men delivered 2 semen samples prior to treatment start and serum RANKL and OPG were measured concomitantly with reproductive and calciotropic hormones at baseline (Supplementary Table 5). In total 22 men were screened, and 12 men were included. All men received calcium and vitamin D supplementation prior to treatment. After treatment start all 12 men were requested to deliver semen and blood samples at day 5, 20, 40, 80, 120, and 180. The early time points were included to avoid missing a putative anti-apoptotic effect due to the fast response of RANKL inhibition observed in human ex vivo models and wild-type mice.

Recruitment	All included men were patients or seen at the department for andrological work up. There will be a selection bias because many of these men have strong desire to become fathers that they often take dietary supplements and may change their way of living during the course of infertility.
Ethics oversight	Copenhagen Bone Gonadal study. The study was approved by the regional ethics committee and EMA approval no. 2010-024588-42, H-4-2010-138, and 2010124801. Human tissue samples and sperm function tests: Patients were recruited from Department of Growth and Reproduction, Rigshospitalet, Denmark in accordance with the Helsinki Declaration after approval from the local ethics committee (H-17004362).The intervention study was approved by the local ethics committee (H-15001992) and the Danish Health authorities and was conducted and monitored according to GCP standard after informed consent was obtained from all participants

Note that full information on the approval of the study protocol must also be provided in the manuscript.

Clinical data

Policy information about clinical studies

All manuscripts should comply with the ICMJE guidelines for publication of clinical research and a completed CONSORT checklist must be included with all submissions.

Clinical trial registration	Copenhagen Bone Gonadal Study NCT01304927 and TNFSF11 Inhibition and Fertility: a Prospective intervention Study NCT02422108
Study protocol	Clinicaltrial.gov but also uploaded here.
Data collection	2011-2017. Patients were recruited from the andrology section at University Department of Growth and Reproduction, Rigshospitalet, Denmark.
Outcomes	The main Primary and secondary outcomes: Semen quality and reproductive hormone analysis were conducted at the department of growth and reproduction using validated and accredited technology. Semen samples were delivered in an adjacent room in the out-patient clinic and information on duration of abstinence, fever, and spillage was obtained. The two semen samples were delivered 10-16 days apart prior to treatment start and analysis was conducted exactly as described in detail previously 11, 44. Briefly, semen volume was determined by weighing, sperm concentration was determined using a Bürker-Türk hemocytometer, and total sperm count was calculated. Sperm morphology was assessed according to strict criteria on Papanicolaou-stained smears. Sperm motility classified as progressive motile (WHO class A+B), nonprogressive motile (class C), or immotile (class D) was determined in duplicate at two times and presented as AB or ABC motility. Spermatozoa DNA fragmentation was investigated at baseline and 20 and 120 days after intervention at SPZ Laboratory (Copenhagen, Denmark).<br><br>Fasting serum samples and seminal fluid were analyzed at all timepoints using validated methodology as described in Supplementary Table 9. Fasting blood samples were collected between 8:00 and 10:00 AM. Serum was analyzed immediately for calcium (total and ionized), phosphate and PTH. LH and FSH using Time-resolved immuno-fluorometric assay Delfia; Wallac, Turku, Finland with CV of 6 and 4%. Inhibin B Two-sided enzyme linked immunoassay Inhibin B genII, Beckman Coulter, USA A81301 CV 11%. AMH Enzyme immunometric assay Immunotech, Beckman Coulter A79765 8%
